# Human influence on sub-regional surface air temperature change over India

**DOI:** 10.1038/s41598-018-27185-8

**Published:** 2018-06-12

**Authors:** R. Dileepkumar, Krishna AchutaRao, T. Arulalan

**Affiliations:** 1 Centre for Atmospheric Sciences, Indian Institute of Technology Delhi, New Delhi, India; 2 National Centre for Medium Range Weather Forecasting, Noida, India

## Abstract

Human activities have been implicated in the observed increase in Global Mean Surface Temperature. Over regional scales where climatic changes determine societal impacts and drive adaptation related decisions, detection and attribution (D&A) of climate change can be challenging due to the greater contribution of internal variability, greater uncertainty in regionally important forcings, greater errors in climate models, and larger observational uncertainty in many regions of the world. We examine the causes of annual and seasonal surface air temperature (TAS) changes over sub-regions (based on a demarcation of homogeneous temperature zones) of India using two observational datasets together with results from a multimodel archive of forced and unforced simulations. Our D&A analysis examines sensitivity of the results to a variety of optimal fingerprint methods and temporal-averaging choices. We can robustly attribute TAS changes over India between 1956–2005 to anthropogenic forcing mostly by greenhouse gases and partially offset by other anthropogenic forcings including aerosols and land use land cover change.

## Introduction

**Figure 1:**
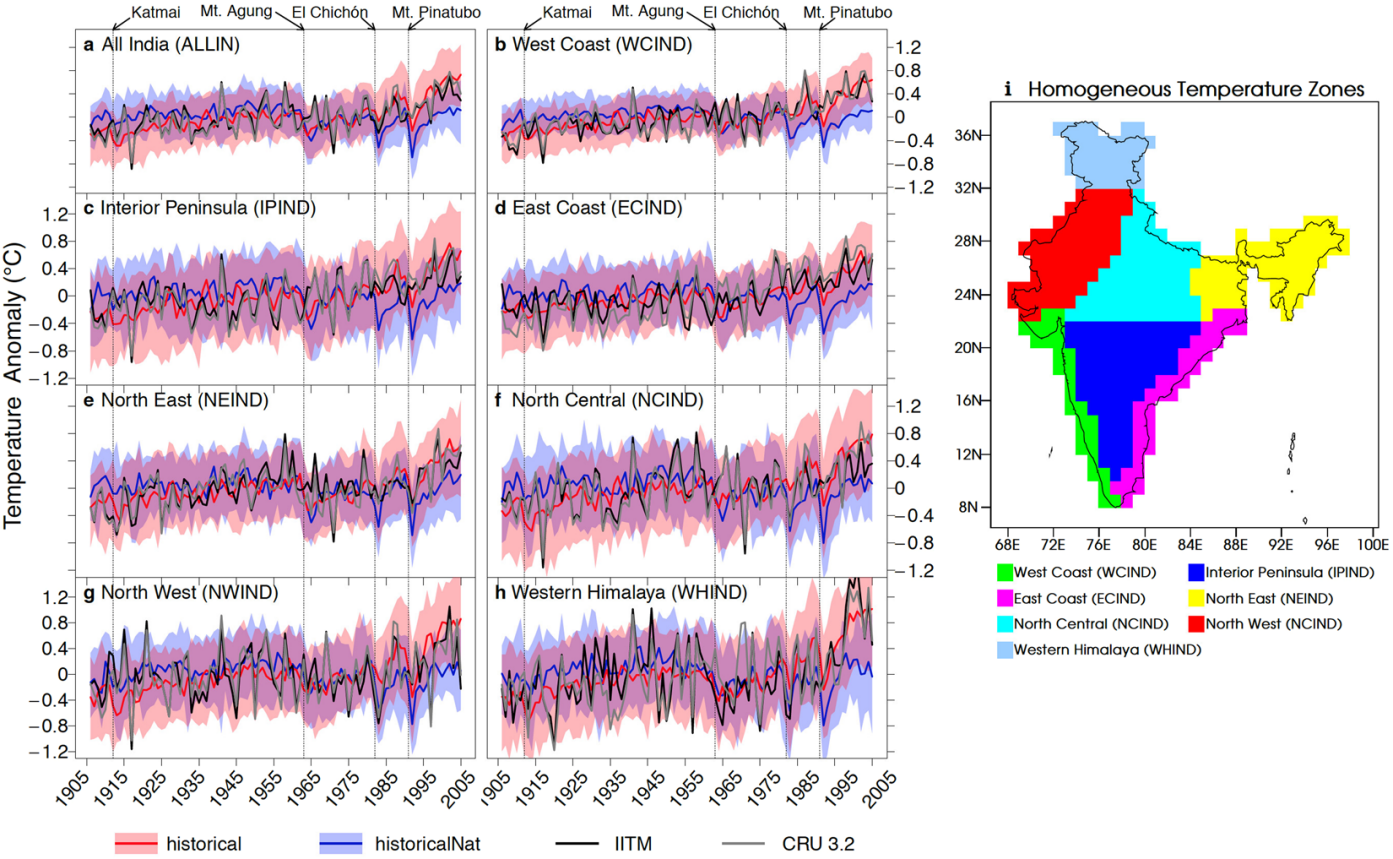
Annual Mean Temperature Anomaly (from 1906–2005 mean) in the observed datasets; IITM (black) and CRU (gray), and in the historical (red) and historicalNat (light-blue) simulations with their 5–95% confidence bounds (calculated as in Jones *et al*. ref.^21^) for (**a)** All India (ALLIN), and the individual homogeneous zones (**b)** West Coast (WCIND), (**c)** Interior Peninsula (IPIND), (**d)** East Coast (ECIND), (**e)** North East (NEIND), (**f)** North Central (NCIND), (**g)** North West (NWIND), and (**h)** Western Himalaya (WHIND). The vertical lines in panels a-h indicate timings of major volcanic eruptions during the 1906–2005 period.The demarcation of homogeneous temperature zones (based on ref.^14^) is shown in (**i**). The map was generated using UVCDAT 2.12 (https://uvcdat.llnl.gov/).

Despite the problems of attributing changes at regional scales, numerous studies provide evidence of an anthropogenic influence on surface temperature at continental and subcontinental scales^1–7^. Temperature changes over the Indian subcontinent during the 20th Century have been documented^8–10^ but have not been subject to formal D&A analysis. The Indian subcontinent contains many sub-regions with vastly different climatic conditions and each of these have witnessed changes to different extents. It is critical for the mitigation and adaptation policy responses of a large developing economy with a population of more than a billion to be informed of the extent to which anthropogenic and natural factors have caused the observed climate changes. In this study we attempt to detect statistically significant changes in surface air temperature (TAS) over sub-regions of India and when detected, quantify contributions of natural (NAT) and anthropogenic (ANT) forcings using formal D&A methods^11^. We also examine whether the anthropogenic forcings due to Greenhouse Gases (GHG) can be isolated from Other Anthropogenic (OA) factors. We use climate model simulations that include individual and combinations of forcings as prescribed in the standard experimental protocols of the Coupled Model Intercomparison Project Phase-5 (CMIP5)^12^. We also use two publicly available long-term datasets of observed temperature over India; i) the CRU-3.22 gridded temperature dataset (0.5° × 0.5° resolution) available from 1901 to 2013^13^, and ii) a monthly mean temperature time-series from 1901 to 2007 for seven “homogeneous temperature zones” obtained from the Indian Institute of Tropical Meteorology (IITM)^14^. The IITM dataset includes observations from many more stations than the CRU dataset over the Indian region. The Indian region (ALLIN) is further divided into seven homogenous temperature zones West Coast (WCIND), East Coast (ECIND), Interior Peninsula(IPIND), North East (NEIND), North Central(NCIND), North West (NWIND), and Western Himalaya (WHIND) based on geographical, topographical and climatological features. The CRU data is regridded to a 1° × 1° grid and masked over each homogeneous temperature zone (see Fig. 1i) before being spatially averaged to produce a monthly time-series analogous to the IITM data. The monthly mean TAS data from the piControl’, ‘historical’, ‘historicalNat’, ‘historicalAnt’, ‘historicalGHG’, and ‘historicalAA’ experiments were obtained from 7 models in the CMIP5 database and treated in the same way as the CRU data (see Table S1 and details of data processing in Supplementary Information). Figure 1a–h shows the annual mean anomaly time series for each of the 8 regions from the two observed datasets as well as the historical and historicalNat simulations (5–95% confidence intervals shown in pink and light-blue shading respectively). The historical and historicalNat simulations are similar for most of the record but diverge from each other after about 1990. Clearly seen in both observations and in the model simulations are the sharp decreases in temperature resulting from volcanic eruptions of the 20^th^ Century.

## Results

### Comparison of observed and model datasets

**Figure 2:**
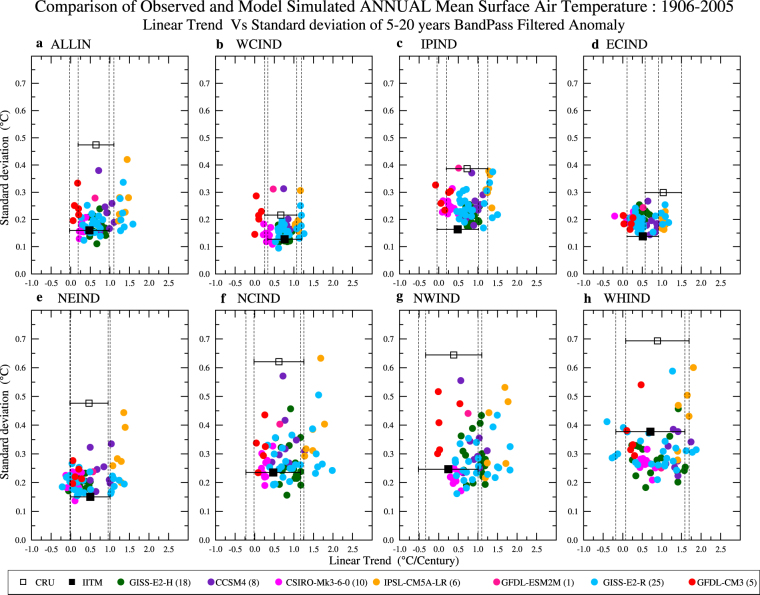
Comparison of observed and modelled linear trend of unfiltered TAS anomaly and standard deviation of 5–20 year bandpass filtered detrended TAS anomaly from CMIP5 historical simulations for the 1906–2005 period over (**a)** ALLIN, (**b)** WCIND, (**c)** IPIND, (**d)** ECIND, (**e)** NEIND, (**f)** NCIND, (**g)** NWIND, and (**h)** WHIND. The numbers inside the brackets next to the model names indicate the total number of realisations used in this study. The trend uncertainty (5–95%) for observations was calculated as in ref.^30^.

We first examine the suitability of the available models to carry out D&A by comparing the decadal-frequency variability simulated by individual model historical experiment realisations with observations. The individual monthly anomaly time series for 1906–2005 were filtered with a 5–20 year band-pass filter (see details in Supplementary material) and the standard deviation of filtered anomaly for ALLIN and the seven homogeneous regions is plotted against the linear trend of the unfiltered time series (Fig. 2). The linear trend for each of the observations with its 5–95% uncertainty range shows that the two datasets are in very good agreement for all the regions except ECIND (Fig. 2d). The CRU time series consistently shows much higher variability than the IITM data in all regions though the standard deviations in the two coastal regions ECIND and WCIND are more in agreement. The discrepancies are mainly due to the larger number of stations included in the IITM dataset and possibly also differences in the homogenization and area averaging/gridding procedures. We note that these differences in variability between the two datasets could have a bearing on the results of D&A analysis. In terms of the variability, most model simulations are clustered around the IITM value in all the regions. The WHIND region (Fig. 2h) stands out as the one region where model simulated variability is lower than observed for most models and to a lesser extent the NCIND and NWIND regions where the GISS-E2-R, GISS-E2-H and CSIRO-MK3-6.0 have lower than observed variability. Taylor Diagrams^15^ comparing CRU and the different experiments and realizations against the IITM reference dataset are shown in Fig. S1 (Supplementary Information). Among the five experiments, the historicalNat simulations are least correlated with observations and the historical and historicalGHG simulations have the highest correlation with observations. The standard deviations in this figure reveal that historicalNat simulations have less than observed variability whereas historicalAA, historicalAnt, historical, and historicalGHG simulations have higher standard deviation than observations. Note that this standard deviation includes variations on all time-scales and trends in the data as they were computed using unfiltered annual mean time-series. On the whole given the small areas of the regions, the CMIP5 simulations perform quite well and the realistic simulation of low-frequency variability is encouraging for carrying out the D&A analysis. While carrying out the D&A analysis using individual models may give different results, we include all the models in our analysis by averaging across models (after averaging each across realisations) thereby giving each model equal weightage rather than weighting each realisation equally (see Supplementary Information for details).

In order to examine the sensitivity of our results to D&A methodology, we used three variants of regression based optimal fingerprint approaches in our analysis of TAS over the Indian region - Ordinary Least Squares (OLS)^16^, Total Least Squares (TLS)^17^, and Regularized Optimal Fingerprinting (ROF)^18^. In all three methods we regress the observations on to the model simulated signals. We calculate the scaling factors for time-series of individual regions and for a combined “All Region” where we retain the spatial information from the seven homogeneous zones (See Supplementary Information for further details). Note that “All Region” is different from ALLIN we have used previously to denote a spatial average over India. We estimate these regressions separately for three time periods (the 50-year periods 1906–1955 and 1956–2005, and the full 100-year period 1906–2005) and two averaging time-lengths (non-overlapping decadal and pentadal averages).

### The two-signal analysis: Natural and Anthropogenic Forcings

**Figure 3:**
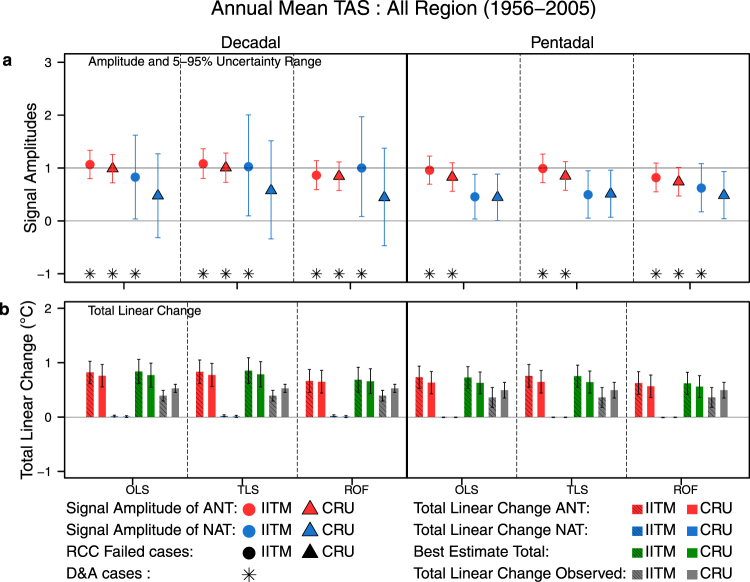
(**a)** The two signal scaling factors and their 5–95% uncertainty levels for “All Region”, estimated for Decadal and Pentadal annual averages between 1956–2005 using OLS, TLS, and ROF methods. (**b)** Best estimate and 5–95% uncertainty range of individual forcing contributions and total linear change of “All Region” annual mean temperature (°C) for 1956–2005 shown for Pentadal and decadal analysis. Best estimates are shown with central 90% uncertainty range. The two solid black symbols indicate cases where Residual Consistency Check (RCC) fails for the corresponding observed datasets and the asterisk indicates cases where the influence of a particular forcing has been detected. It may be noted that no cases fail the RCC. Observed trend uncertainties (5–95%) were calculated as in ref.^30^.

In order to estimate the contribution of Natural and Anthropogenic forcings to changes in TAS, we implemented a two-signal optimal fingerprinting analysis in which observations are regressed on to historicalAnt and historicalNat^19^ to yield estimates of the scaling factors ${\beta }_{{ANT}}$ and ${\beta }_{{NAT}}$ with their 5–95% uncertainty ranges (See Data and Methods section and Supplementary Information for further details). In Fig. 3a the “All Region” scaling factors for annual mean temperatures from three different regression methods, two observational data sets, and two averaging time-lengths are shown for the 1956–2005 period. We note that for both the decadal and pentadal analysis, the ANT signal amplitudes from all three methods for both the CRU and IITM reference data are consistent with unity. In all three decadal analyses the amplitude of the NAT signal is consistent with unity for the IITM dataset but consistent with zero for CRU. However, for the pentadal analysis, the NAT signal is consistent with unity only for the ROF method using IITM. The best estimate scaling factors for ANT and NAT in two-signal decadal analyses of “All Region”, together with their marginal confidence intervals and joint confidence regions for each of the observed data sets are shown in Fig. S2 (Supplementary Information). The ANT scaling factors are consistent with one for OLS, TLS, and ROF methods while the NAT scaling factors are not significantly different from zero. This indicates that the simulated ANT response is consistent with observed changes while the NAT forcings are not substantially contributing to observed changes.

In Fig. 3b the linear trends of scaled reconstructed TAS over the 1956–2005 period are shown, revealing that the observed and reconstructed total linear change are in good agreement in the Decadal ROF and all the Pentadal analyses. Furthermore, the reconstructed trends are almost entirely as a result of the ANT forcing with negligible contribution of NAT forcings to the trends. Examining the trends from the decadal ROF analysis over the 1956–2005 period gives attributable trends (in °C per 50 years given as 5–95% range) for ANT of (0.44, 0.86), for NAT of (−0.01, 0.03), and the best estimate reconstructed trend of (0.43, 0.89) with the CRU observed trend of (0.45, 0.60). For the same period, pentadal averages reveal attributable trends for ANT of (0.36, 0.77) and NAT of (0.0, −0.01) with the CRU observed trend of (0.35, 0.64). Table 1 shows the trends attributable to ANT and NAT forcings for the different time-periods, temporal averaging lengths, and observed trends for both observations and clearly reveals that where analysis period includes the 1956–2005 half-century, when ANT forcing is strongest, ANT response dominates over NAT response.

**Table 1:** Observed and attributable trends (°C per period length given as 5–95% ranges) over “All Region” from the two-signal analysis of TAS change.

Period	Ref. Dataset	Decadal Trends					Pentadal Trends				
		Observed		OLS	TLS	ROF	Observed		OLS	TLS	ROF
1956–2005	IITM	(0.30, 0.49)	B. Est.	(0.62, 1.06)	(0.62, 1.09)	(0.46, 0.92)	(0.18, 0.54)	B. Est.	(0.53, 0.93)	(0.55, 0.96)	(0.42, 0.82)
			ANT	(0.61, 1.03)	(0.62, 1.05)	(0.45, 0.88)		ANT	(0.53, 0.94)	(0.55, 0.97)	(0.42, 0.83)
			NAT	(0.00, 0.03)	(0.00, 0.04)	(0.00, 0.04)		NAT	(−0.01, 0.00)	(−0.01, 0.00)	(−0.01, 0.00)
	CRU	(0.45, 0.60)	B. Est.	(0.55, 0.99)	(0.56, 1.02)	(0.43, 0.89)	(0.35, 0.64)	B. Est.	(0.43, 0.83)	(0.44, 0.85)	(0.36, 0.76)
			ANT	(0.55, 0.97)	(0.56, 0.99)	(0.44, 0.86)		ANT	(0.43, 0.84)	(0.44, 0.86)	(0.36, 0.77)
			NAT	(0.00, 0.03)	(0.00, 0.03)	(−0.01, 0.03)		NAT	(−0.01, 0.00)	(−0.01, 0.00)	(−0.01, 0.00)
1906–2005	IITM	(0.29, 0.53)	B. Est.	(0.61, 0.86)	(0.63, 0.87)	(0.40, 0.59)	(0.23, 0.60)	B. Est.	(0.56, 0.85)	(0.52, 0.78)	(0.32, 0.56)
			ANT	(0.63, 1.00)	(0.66, 1.04)	(0.41, 0.72)		ANT	(0.55, 0.93)	(0.52, 0.89)	(0.33, 0.67)
			NAT	(−0.14, −0.02)	(−0.17, −0.03)	(−0.13, −0.01)		NAT	(−0.08, 0.02)	(−0.11, 0.00)	(−0.10, −0.01)
	CRU	(0.41, 0.68)	B. Est.	(0.73, 0.98)	(0.77, 1.01)	(0.54, 0.73)	(0.36, 0.73)	B. Est.	(0.56, 0.85)	(0.63, 0.91)	(0.43, 0.67)
			ANT	(0.70, 1.08)	(0.75, 1.13)	(0.53, 0.84)		ANT	(0.54, 0.93)	(0.59, 0.99)	(0.44, 0.77)
			NAT	(−0.09, 0.03)	(−0.12, 0.02)	(−0.11, 0.02)		NAT	(−0.08, 0.02)	(−0.08, 0.04)	(−0.10, −0.01)
1906–1955	IITM	(0.21, 0.28)	B. Est.	(−0.22, 0.71)	(−0.36, 0.88)	(−0.53, 0.90)	(0.20, 0.35)	B. Est.	(−0.13, 0.58)	(−0.23, 0.79)	(−0.29, 0.73)
			ANT	(−0.16, 0.38)	(−0.27, 0.44)	(−0.30, 0.51)		ANT	(0.03, 0.45)	(0.06, 0.67)	(−0.06, 0.55)
			NAT	(−0.06, 0.33)	(−0.09, 0.44)	(−0.23, 0.39)		NAT	(NA, NA)	(NA, NA)	(NA, NA)
	CRU	(0.25, 0.33)	B. Est.	(−0.33, 0.60)	(−0.43, 0.70)	(−0.45, 0.77)	(0.25, 0.40)	B. Est.	(−0.22, 0.50)	(−0.32, 0.64)	(−0.32, 0.71)
			ANT	(−0.27, 0.28)	(NA, NA)	(NA, NA)		ANT	(−0.16, 0.26)	(−0.22, 0.36)	(−0.25, 0.37)
			NAT	(−0.07, 0.32)	(−0.07, 0.41)	(−0.09, 0.44)		NAT	(−0.06, 0.24)	(−0.11, 0.28)	(−0.07, 0.34)

Results are shown for the three periods analysed 1956–2005, 1906–2005, and 1906–1955. The ANT and NAT attributable trends are shown along with the Best Estimate from the two forcings. Observed trends and 5–95% confidence interval are calculated as in ref.^30^. The cells marked (NA, NA) represent cases where the residual consistency check (RCC) fails or there was no detection (β < 0).

When we analyse the individual homogeneous zones, we can detect the influence of ANT forcing over the WCIND, IPIND, and ECIND regions for both observational datasets. Additionally, the magnitude of observed trend is almost entirely due to ANT trend in each of these cases (see Fig. S3a in the Supplementary Information). The Western Himalayan region (WHIND) has seen the largest rates of warming among all the regions and although responses to ANT and NAT forcings are detected (only for the IITM dataset), ANT forcing contributes to nearly all of the trend. These are robust except for the decadal analysis using the ROF method which fails the residual consistency check. The Residual Consistency Check (RCC; see Supplementary Material for details) - carried out for all 3 methods in each of the individual regions and seasons - tests the hypothesis that model simulated internal variability is consistent with observed (similar to what Fig. 2 shows). Unlike the “All Region” case (which includes the spatial dimension also) the individual regions only have 5 decadal time points leading to a situation where there is very little information left to estimate the residual’s variance. When we consider the 100-year long 1906–2005 period also (see Fig. S3b in Supplementary Information) we see similar detection of ANT signals in the WCIND, IPIND, ECIND, and WHIND regions. Between 1906–1955 (see Fig. S3c in Supplementary Information), the observed trends in each of the regions are smaller and we were unable to attribute these changes to either NAT or ANT forcings. It is therefore clear that most of the changes in annual mean TAS seen in the 100 year period have occurred over the second 50-year period (1956–2005), and that ANT forcing is responsible for almost all of the change.

**Figure 4:**
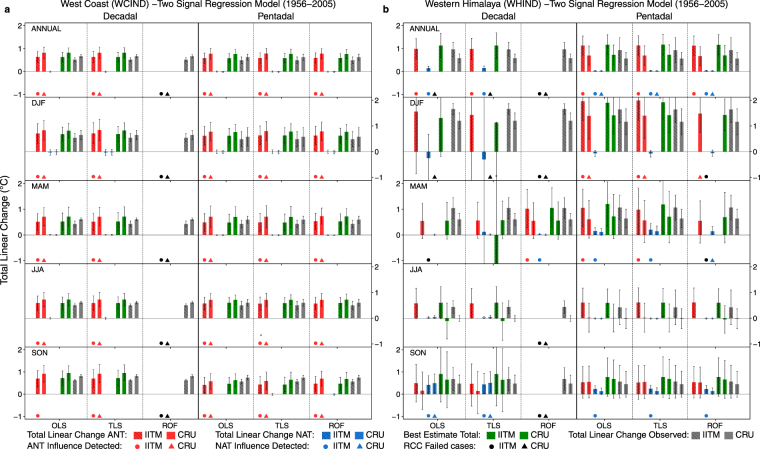
The two-signal total linear change of observed and scaled reconstructed simulated forcings of TAS using best signal amplitude ($\beta$) from OLS, TLS and ROF methods for Pentadal and Decadal analysis of annual and seasonal means over 1956–2005 period for (**a)** West Coast (WCIND) and (**b)** Western Himalayas (WHIND). The hatched bars and non-hatched bars represent the analysis using IITM and CRU as observed dataset respectively. Red(ANT), blue(NAT), green(Best Estimate), and gray(Observed) bars represent the total linear change of simulated response and observations. The cases where effects of ANT (NAT) forcings were detected are marked using red (blue) symbols with circles representing IITM dataset and triangles representing CRU. The black symbols represent cases where the residual consistency check (RCC) failed. Where bars are missing, either the RCC fails for the corresponding signal or there was no detection (β < 0). Observed trend uncertainties (5–95%) were calculated as in ref.^30^.

We now focus our attention on WCIND and WHIND regions to delve into the seasonality of changes. Figure 4a shows that, over the WCIND region, ANT forcing is detected for each of the seasons and annual mean irrespective of observed dataset used. Moreover, the effects of NAT forcings are not detected. This is robust across the different methods - except the decadal ROF analysis where the RCC fails for reasons noted earlier. The effects of ANT forcings are detected in the DJF season over the WHIND in the pentadal analysis (Fig. 4b). The effects of NAT forcings are also detected but do not make a significant contribution to the annual mean trends and contribute to a slight warming during the MAM and SON seasons. This is also revealed in the reconstructed pentadal time series (see Fig. S4 in Supplementary Information). The NEIND, NCIND, and NWIND regions have smaller observed trends when compared to the other regions and we were able to detect the ANT forcing effects only when using the CRU dataset (see Fig.S3a in Supplementary Information).

### Decomposing the Anthropogenic forcing

Since anthropogenic forcings include the effects of greenhouse gases, anthropogenic aerosols, and land-use land-cover changes (LULC) we explore the possibility of decomposing the ANT signal into these signals. The treatment of anthropogenic aerosols and LULC are quite different from model to model with some models not including the indirect effects of aerosols (see Table S1 in the Supplementary Information for details). One of the models considered (CSIRO-MK3-6-0) did not include changes in LULC and neither the CMIP5 experimental setup nor the specific models chosen included an LULC only set of runs. It is not always possible to decompose the signals as a simple linear addition of forcings - for instance, the combination of greenhouse gases and indirect effects of aerosols^20^. We therefore decompose the ANT signal into a GHG forcing driven signal and an “Other Anthropogenic” (OA) signal (as in ref.^21^) which subsumes all non-GHG anthropogenic forcings under the “OA” forcing. For this the historical, historicalGHG, and historicalNAT simulations were used to construct the transformation equation for deducing the scaling factors ${\beta }_{{GHG}}$, ${\beta }_{N{AT}}$, and ${\beta }_{{OA}}$ corresponding to GHG, NAT, and OA forcings respectively (See Data and Methods section for details).

**Figure 5:**
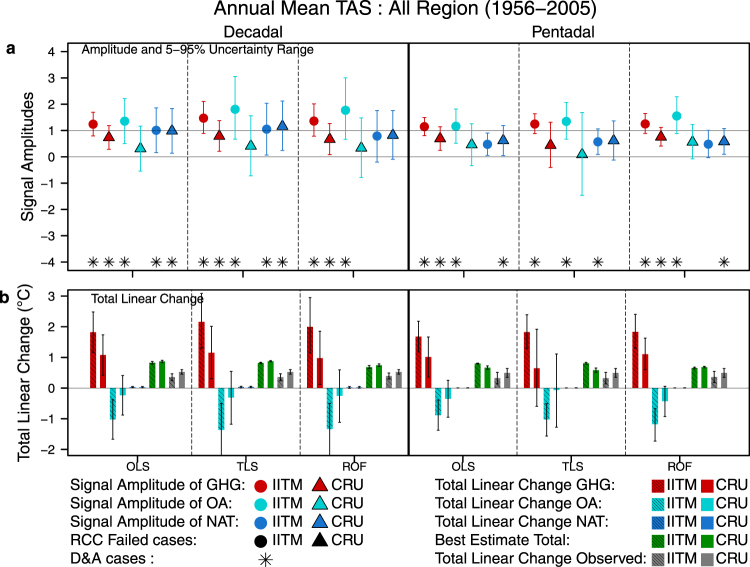
(**a)** The three signal scaling factors and their 5–95% uncertainty levels for “All Region” estimated for both decadal and pentadal annual averages between 1956–2005 using OLS, TLS, and ROF methods. (**b)** Best estimate and 5–95% uncertainty range of individual forcing contributions and total linear change of “All Region” annual mean temperature (°C) for 1956–2005 shown for pentadal and decadal analysis. Best estimates are shown with central 90% uncertainty range. The two solid black symbols indicate cases where Residual Consistency Check (RCC) fails for the corresponding observed datasets and the asterisk indicates cases where the influence of a particular forcing has been detected. It may be noted that no cases fail the RCC. Observed trend uncertainties (5–95%) were calculated as in ref.^30^.

Figure 5 shows the results of the three-signal analyses of annual mean TAS over “All Region” for the 1956–2005 period. The scaling factors in Fig. 5a show that the influence of GHG forcing can be detected over “All Region” for both observations and in all cases except pentadal TLS analysis with CRU observations. The effect of OA forcings can be detected in all cases where IITM observations are used but in none of the cases when CRU is used. Although the analysis reveals a detectable NAT signal for many cases, the trend contribution from NAT is minimal as seen in Fig. 5b. The dominant signal in all the cases seen is the positive trend from GHG forcing with the negative trend produced by OA forcings counteracting it. This results in a net positive trend in the reconstructed TAS that closely matches the observed trends. The uncertainty range of scaling factors and trends for GHG forcing are smaller than those for OA and NAT forcings. Moreover the amplitudes of the two anthropogenic signals (GHG & OA) are highly correlated as evidenced by the strong tilt in the marginal uncertainty ellipse (see Fig. S5 in Supplementary Information).

When we analyse the “All Region” changes for individual seasons, there are numerous cases where we detect the effects of GHG and OA forcings (Fig. S6a in Supplementary Information). However, these are not consistent across seasons or observational datasets. The effects of NAT forcings are detected in a handful of cases but do not contribute significantly to the trends. The 100-year 1906–2005 period on the other hand provides the strongest evidence of the combined influence of the GHG and OA forcings irrespective of observational dataset used, particularly through the decadal analysis for all the seasons and the pentadal ROF analysis of the Annual, DJF, MAM, and SON seasons (see Fig. S6b in Supplementary Information). The NAT forcings are also clearly detected in the MAM and JJA (decadal analysis only) seasons although they contribute only a very small negative trend. Over the 1906–1955 period, the small observed trends were found to be due to NAT forcings in the MAM season but are otherwise not detected in any season (Fig. S6c).

In the analysis of individual homogeneous regions, we detect the effects of GHG forcing in the WCIND region with the pentadal analysis for all seasons except the MAM season with some sensitivity to observational dataset used (Fig. S7a in Supplementary Information). Barring one case, the effects of OA are not detected at all in this region. Over the WHIND region, the effects of GHG and OA forcings are detected in the DJF season for pentadal analysis only (Fig. S7b in Supplementary Information). The WHIND region also indicates a detectable influence of NAT forcings in the pentadal analysis of Annual, MAM and SON seasons which results in a very small negative trend. The MAM season decadal analysis shows clear detection of the influence of GHG, OA, and NAT forcings for the WHIND. Analysis of the Annual mean TAS in the ECIND region shows that the effect of GHG forcings is detected, but not that of OA or NAT (not shown). In the IPIND, NEIND, NCIND, and NWIND regions we were not able to detect the effects of GHG or OA forcings except in one or two cases.

Recognising that the forcings under “OA” predominantly include the effects of anthropogenic aerosols and LULCC, the inclusion/omission of indirect effects of aerosols is likely an important factor that determines the amount of cooling produced by aerosol effects^21^. To investigate this further, we ran the three signal analysis with only the subset of models that include the indirect effects of models and land use changes (i.e by dropping the GFDL-ESM2M, CCSM4, and CSIRO-MK3-6-0 models from our analysis). The results thus obtained were not very different from the analysis that included all the models except for a reduction in the uncertainty ranges around the $\beta$ values.

## Conclusions

Using three regression based optimal fingerprint methods, two observed datasets, and two temporal-averaging choices we are able to robustly attribute TAS changes over India between 1956–2005 and 1906–2005 to anthropogenic forcing. The contributions of natural forcings to the observed trend during 1956–2005 period is minimal with a comparatively larger contribution during 1906–1955. We also find that the observed increases in TAS over WCIND, IPIND, ECIND, and WHIND cannot be explained by natural forcings or internal climate variability alone. The largest warming rates are seen in the Western Himalayas - a mountainous region that is likely experiencing elevation enhanced warming^22^. This has implications for the health of glaciers in this region with continued anthropogenic forcing leaving them more prone to mass loss and resulting downstream effects^23^. The influence of anthropogenic forcing is also detected in NEIND, NCIND, and NWIND, but only with the CRU dataset. However, the two observational datasets are different in the number of observing stations they include and this influences results to some extent. Using a three-signal analysis, we find that GHG forcing is the dominant contributor to the positive trends over India with OA forcings exerting a cooling influence. Over the individual sub-regions however, we were not able to consistently detect the influence of GHG and OA. The influence of aerosols^24^ and land use (that are the main constituents of OA) cannot be separated using these methods which assume that signals can be decomposed as a simple linear addition of forcings. The impact of observational uncertainty (to the extent evident in the two datasets used) is clearly seen in our results for individual regions. Concerted efforts to quantify observational uncertainty at these smaller spatial scales are necessary to have better confidence in D&A results and also to enable climate model improvements^25^.

## Data and Methods

### Observed data

We used surface air temperature (TAS) data from the CRU 3.22 and IITM observational datasets. The CRU- 3.22^13^ is a gridded temperature dataset at 0.5 × 0.5 resolution downloaded from https://crudata.uea.ac.uk/cru/data/hrg/cru_ts_3.22/. Spatially averaged monthly temperature data (1906–2005) for seven homogeneous temperature zones^14^ and ALLIN region were downloaded from http://www.tropmet.res.in/static_page.php?page_id = 54.

### Model data

We utilize climate model output from the Fifth Phase of the Coupled Model Intercomparison Project CMIP5^12^. While output from nearly 60 coupled atmosphere-ocean general circulation models (AOGCMs) are available in the CMIP5 archives, we utilize output from models that performed piControl (Pre-Industrial control), historicalGHG (GHG forcings only), historicalNat (Natural forcings only), historicalAA (forcings due to anthropogenic aerosols only) and historicalAnt (all forcing due to anthropogenic activities) and historical (combination of all forcings) experiments. The AOGCMs used in this study span a wide range of resolutions and sophistication of physical modeling. Each model experiment may have more than one ensemble member typically generated by initializing a set of runs with different, but equally realistic initial conditions that are distinguished by a unique realization number. We use the subset of models that performed all the experiments listed above and whose output fields included surface air temperature (TAS) and -although these were not analyzed for this study, minimum and maximum surface air temperature (TASMIN and TASMAX respectively) - in order to avoid changing sample sizes. Table S1 in the Supplementary Information shows details of the models, experiments, and realizations used in this study.

### Details of Detection & Attribution methods used

Optimal fingerprinting is generalized multivariate regression adapted to the detection of climate change and the attribution of change to externally forced climate change signals^26,27^. We implemented Ordinary Least Squares (OLS)^16^ and Total Least Squares (TLS)^17^ methods to carry out the detection analysis. In both methods observations $Y$ are regressed on to $m$ model simulated signals ${X}_{i}$. The TLS regression model is of the form

1 $${\boldsymbol{Y}}={\sum }_{{\boldsymbol{i}}=1}^{{\boldsymbol{m}}}({{\boldsymbol{X}}}_{{\boldsymbol{i}}}-{{\boldsymbol{v}}}_{{\boldsymbol{i}}}){{\boldsymbol{\beta }}}_{{\boldsymbol{i}}}+{{\boldsymbol{v}}}_{0}$$

where ${v}_{0}$ represents the residual variability that is generated internally in the climate system and ${\upsilon }_{i}$ is an additional noise term included to account for the finite model sample ${X}_{i}$ (see ref.^17^). In the OLS case we assume the noise *v*_*i*_ = 0. We assert detection when ${\beta }_{i}$ is significantly greater than zero and if it is consistent with unity we attribute observed changes to forcing $i$ (such cases are denoted by asterisks in Figs 3 and 5). We used two noise covariance matrices ${C}_{N1}$ and ${C}_{N2}$ for estimating the signal amplitude ${\beta }_{i}$ and for the residual consistency check respectively (see the Supplementary Information for details of constructing ${C}_{N1}$ and ${C}_{N2}$).

In both OLS^16^ and TLS^17^ methods ${C}_{N1}^{-1}$ is constructed from the reduced dimension of EOF and this requires a truncation value $\kappa$. Using the regularization method in ref.^28^, Ribes *et al*. (ref.^18^) developed the Regularized Optimal Fingerprinting (ROF) method that uses an inverse of the covariance matrix avoiding the use of a truncated EOF projection. Using Monte Carlo simulations, Ribes *et al*. (ref.^29^) showed that the ROF method yields more accurate (in terms of the mean squared error) results. We implement the ROF method with Total Least Squares regression. Using these three methods (OLS, TLS, and ROF) we applied two regression based models; two-signal and three-signal, for carrying out the detection and attribution study. The transformation of two- and three-signal regression models are as follows:

### Two Signal Regression Model

In this model we estimate the contribution of natural (NAT) and anthropogenic (ANT) forcings to changes in TAS. The two-signals ${X}_{{ANT}}$ and ${X}_{{NAT}}$ are derived using historicalAnt and historicalNat simulations (see Supplementary Information for further details on simulations) and the corresponding scaling factors (from Eq. 1) are ${\beta }_{{ANT}}$ and ${\beta }_{{NAT}}$.

### Three Signal Regression Model

We analyze the contributions from greenhouse gas forcing (${X}_{{GHG}}$), other non-greenhouse gas anthropogenic forcings (${X}_{{OA}}$) and natural forcings (${X}_{{NAT}}$) with corresponding scaling factors ${\beta }_{{GHG}}$, ${\beta }_{{OA}}$, and ${\beta }_{{NAT}}$. Since we only have the historical, historicalGHG, and historicalNat experiments we deduce the scaling factors from a transformation on the historical, historicalNat, and historicalGHG scaling factors ${\beta }_{{historical}}$, ${\beta }_{{historicalGHG}}$, and ${\beta }_{{historicalNat}}$ as in Tett *et al*. (ref.^19^) and Jones *et al*. (ref.^21^):

2 $$(\begin{array}{c}{{\boldsymbol{\beta }}}_{{\boldsymbol{G}}{\boldsymbol{H}}{\boldsymbol{G}}}\\ {{\boldsymbol{\beta }}}_{{\boldsymbol{O}}{\boldsymbol{A}}}\\ {{\boldsymbol{\beta }}}_{{\boldsymbol{N}}{\boldsymbol{A}}{\boldsymbol{T}}}\end{array})=(\begin{array}{ccc}1 & 1 & 0\\ 0 & 1 & 0\\ 0 & 1 & 1\end{array})(\begin{array}{c}{{\boldsymbol{\beta }}}_{{\bf{h}}{\bf{i}}{\bf{s}}{\bf{t}}{\bf{o}}{\bf{r}}{\bf{i}}{\bf{c}}{\bf{a}}{\bf{l}}{\bf{G}}{\bf{H}}{\bf{G}}}\\ {{\boldsymbol{\beta }}}_{{\bf{h}}{\bf{i}}{\bf{s}}{\bf{t}}{\bf{o}}{\bf{r}}{\bf{i}}{\bf{c}}{\bf{a}}{\bf{l}}}\\ {{\boldsymbol{\beta }}}_{{\bf{h}}{\bf{i}}{\bf{s}}{\bf{t}}{\bf{o}}{\bf{r}}{\bf{i}}{\bf{c}}{\bf{a}}{\bf{l}}{\bf{N}}{\bf{a}}{\bf{t}}}\end{array})$$

Further details on the implementation can be found in the Supplementary Information.

### Data availability

Data for CMIP5 models are available at https://esgf-node.llnl.gov/projects/esgf-llnl/. The CRU- 3.22 dataset is available from https://crudata.uea.ac.uk/cru/data/hrg/cru_ts_3.22/. The IITM dataset are available from http://www.tropmet.res.in/static_page.php?page_id = 54. The datasets generated during the current study are available from the corresponding author (KAR) on reasonable request.

## Electronic supplementary material

Supplementary Information
