# Supplementary material for: Human influence on sub-regional surface air temperature change over India
Source: Sci Rep. 2018 Jun 12;8:8967. doi: 10.1038/s41598-018-27185-8 (PMC5997713; doi:10.1038/s41598-018-27185-8)
Supplement: Supplementary file 1 — Supplementary Information [file 41598_2018_27185_MOESM1_ESM.pdf]

Supplementary information

Human influence on sub-regional surface air temperature change over India

Dileepkumar R[1], Krishna AchutaRao[1,*], Arulalan T[1, 2]

[1*] Centre for Atmospheric Sciences, Indian Institute of Technology Delhi, New Delhi, India

[2] National Centre for Medium Range Weather Forecasting, Noida, India

[*] Corresponding Author: akrishna@cas.iitd.ac.in

File Name: HumanInfluenceSubRegionalIndiaTemperatures_SupplementaryInformation.pdf

Contents

Figures

Figure S1. Taylor diagram[1] of annual mean TAS for ALLIN and the seven homogeneous regions for the 1906-2005 period. CMIP5 model experiments historical (All forcings, red), historicalGHG (Greenhouse Gases forcings, blue), historicalAnt (Anthropogenic forcings, pink), historicalAA (Anthropogenic Aerosols, orange) and historicalNat (Natural forcings, green) are compared against IITM (observed)[2] as reference dataset. Each colored symbol represents one model simulation. The CRU 3.22 dataset[3] statistics are also shown as a black triangle providing a measure of observational uncertainty. The brown arcs represent the constant standard deviation curves and dotted purple arcs show the constant centered root mean square error (RMSE). Note that all the statistics shown are calculated with unfiltered time-series data.

Figure S2. Amplitudes with 5-95% marginal confidence intervals (bars) and 90% confidence regions (ellipses) for ANT vs NAT derived from two-signal analysis using **a** OLS, **b** TLS, and **c** ROF methods

using decadal anomalies of annual mean for 1956-2005 period over “All Region”. The red and black colours indicate the analysis using CRU 3.22 and IITM datasets respectively.

Figure S3. The total linear change of observed and scaled reconstructed simulated forcings of TAS using signal amplitude (β) from OLS, TLS and ROF methods for Decadal and Pentadal analysis of Annual Mean TAS for “All Region” and the seven homogeneous regions for **a** 1956-2005, **b** 1906-2005, and **c** 1906-1955. The hatched bars and non-hatched bars represent the analysis using IITM and CRU as observed dataset respectively. Red(ANT), blue(NAT), green(Best Estimate), and gray(Observed) bars represent the total linear change of simulated response and observations. The cases where effects of ANT (NAT) forcings were detected are marked using red (blue) symbols with circles representing IITM dataset and triangles representing CRU. The black symbols represent cases where the residual consistency check (RCC) failed. Where bars are missing, either the RCC fails for the corresponding signal or there was no detection ($\beta < 0$). Observed trend uncertainties (5-95%) were calculated as in supplementary ref. 5.

Figure S4. The OLS best estimate reconstruction of pentadal temperature variations during 1956-2005 for **a** DJF, **b** MAM, **c** JJA, **d** SON, and **e** Annual seasons in the WHIND region (black dashed line) shown along with IITM observed dataset (solid black line), and best-estimate contributions from ANT (magenta) and NAT (green). Shaded region centered on the observations shows the uncertainty range due to internal variability (two-sigma pentadal variability computed from C_{N2}).

Figure S5. Amplitudes with 5-95% marginal confidence intervals (bars) and 90% confidence regions (ellipses) derived from three-signal **a** OLS, **b** TLS, and **c** ROF analysis GHG vs OA, GHG vs NAT and OA vs NAT using pentadal anomaly of annual mean for 1956-2005 period for “All Region”. The red and black colours indicate the analysis using CRU and IITM datasets respectively.

Figure S6. The three-signal total linear change of observed and scaled reconstructed simulated forcings of TAS for “All Region” using best signal amplitude (β) from OLS, TLS and ROF methods for Pentadal and Decadal analysis of annual and seasonal means over **a** 1956-2005, **b** 1906-2005, and **c** 1906-1955.

The hatched bars and non-hatched bars represent the analysis using IITM and CRU as observed dataset respectively. Red(GHG), cyan (OA), blue(NAT), green(Best Estimate), and gray(Observed) bars represent the total linear change of simulated response and observations. The cases where effects of individual forcings were detected are marked using colored symbols with circles representing IITM dataset and triangles representing CRU. The black symbols represent cases where the residual consistency check (RCC) failed. Where bars are missing, either the RCC fails for the corresponding signal or there was no detection ($\beta < 0$). Observed trend uncertainties (5-95%) were calculated as in supplementary ref. 5.

Figure S7. The three-signal total linear change of observed and scaled reconstructed simulated forcings of TAS using best signal amplitude (β) from OLS, TLS and ROF methods for Pentadal and Decadal analysis of annual and seasonal means over 1956 - 2005 period for **a** West Coast (WCIND) and **b** Western Himalayan (WHIND) regions. The hatched bars and non-hatched bars represent the analysis using IITM and CRU as observed dataset respectively. Red(GHG), cyan (OA), blue(NAT), green(Best Estimate), and gray(Observed) bars represent the total linear change of simulated response and observations. The cases where effects of individual forcings were detected are marked using colored symbols with circles representing IITM dataset and triangles representing CRU. The black symbols represent cases where the residual consistency check (RCC) failed. Where bars are missing, either the RCC fails for the corresponding signal or there was no detection ($\beta < 0$). Observed trend uncertainties (5-95%) were calculated as in supplementary ref. 5.

Figure S8. The Residual Consistency Check (RCC) for **I** pentadal SON season analysis in the NEIND region and **II** pentadal MAM season analysis in the NCIND region over 1956-2005 period and using three signal TLS method. The red circles and whiskers in sub-figures **a**, **b**, and **c** of both I and II represent the "best estimate" and the 5 - 95% range for the estimated amplitude of GHG, OA, and NAT forcings respectively for each number of EOFs retained in the truncation. The red and green dotted lines in panel **d** of I and II represent the 5 -95 % confidence level. The RCC fails in I, and passes in II with

maximum EOF number as 7. For truncations ≤ 7, the Cumulative ratio model/Observed variance (solid blue line) ranges around unity shows that the model variability is consistent with observed, but for greater truncation levels it falls outside the 5-95 % confidence band. The scaling factor corresponding to EOF 7 was selected as the signal amplitude.

Tables

Table S1. CMIP5 models[4] used in Optimal Fingerprint analysis. Model names, data resolution, number of initial condition ensemble members for each experiment and number of years available for each model's pre-industrial control run also listed. The last column shows the forcing factor accounted for in addition the model runs. Y indicates factor included and N indicates factor not included.

Table S2. Area weights for the different homogeneous zones used to compute signal amplitude of "All Regions".

Table S3. Observed and attributable trends (°C per period length given as 5–95% ranges) over "All Region" from the three-signal analysis of TAS change. Results are shown for the three periods analysed 1956-2005, 1906-2005, and 1906-1955. The GHG, OA, and NAT attributable trends are shown along with the Best Estimate from the three forcings. Observed trends and 5-95% confidence interval are calculated as in Santer et al 2000 (ref. 5). The cells marked (NA, NA) represent cases where the residual consistency check (RCC) fails or there was no detection ($\beta < 0$).

Methods Details

1. Band-pass filter details
2. Processing of data
3. Truncation level and Residual Consistency Check (RCC)

Supplementary References

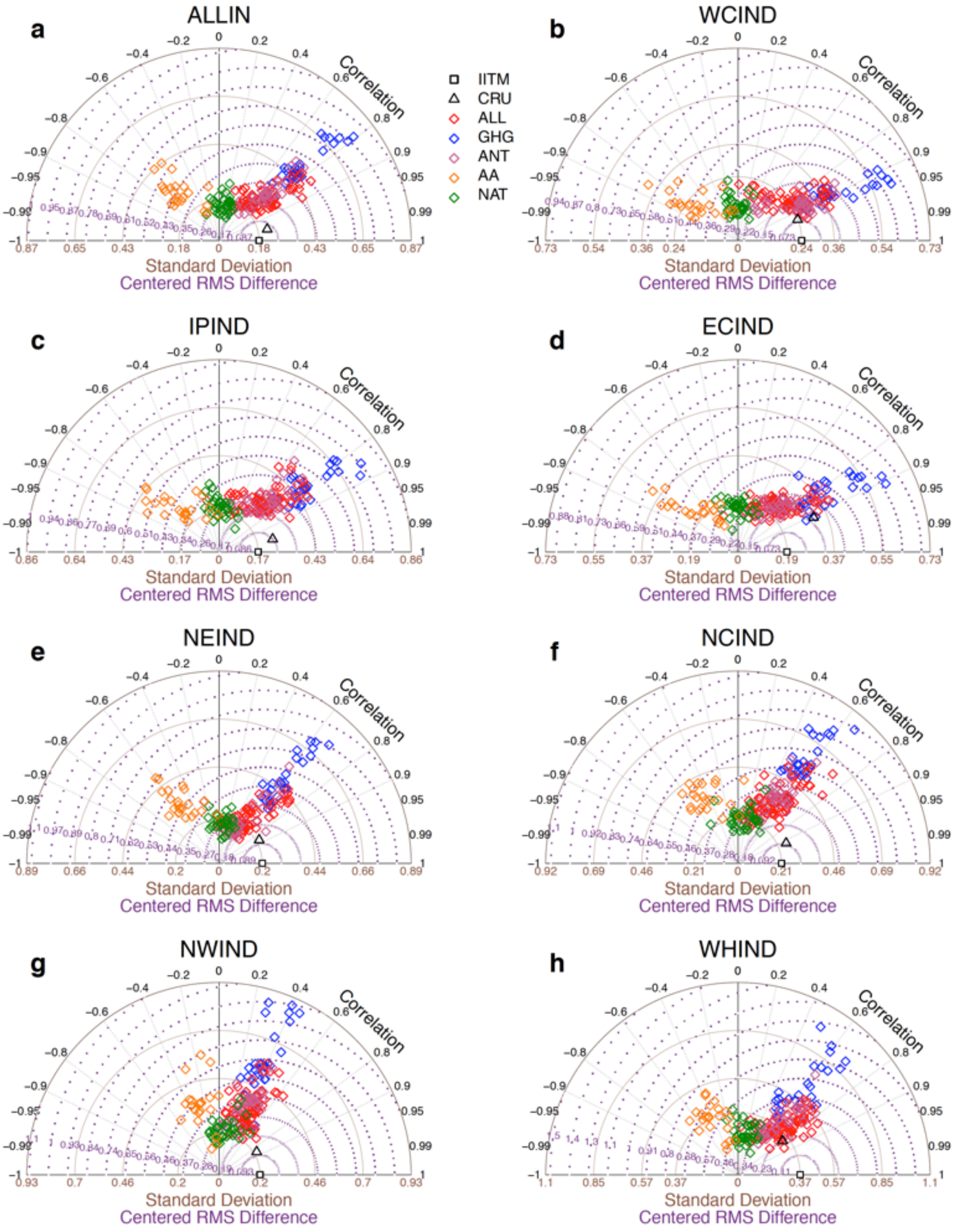

Figure S1. Taylor diagram[1] of annual mean TAS for ALLIN and the seven homogeneous regions for the 1906-2005 period. CMIP5 model experiments historical (All forcings, red), historicalGHG (Greenhouse Gases forcings, blue), historicalAnt (Anthropogenic forcings, pink), historicalAA (Anthropogenic Aerosols, orange) and historicalNat (Natural forcings, green) are compared against IITM (observed)[2] as reference dataset. Each colored symbol represents one model simulation. The CRU 3.22 dataset[3] statistics are also shown as a black triangle providing a measure of observational uncertainty. The brown arcs represent the constant standard deviation curves and dotted purple arcs show the constant centered root mean square error (RMSE). Note that all the statistics shown are calculated with unfiltered time-series data.

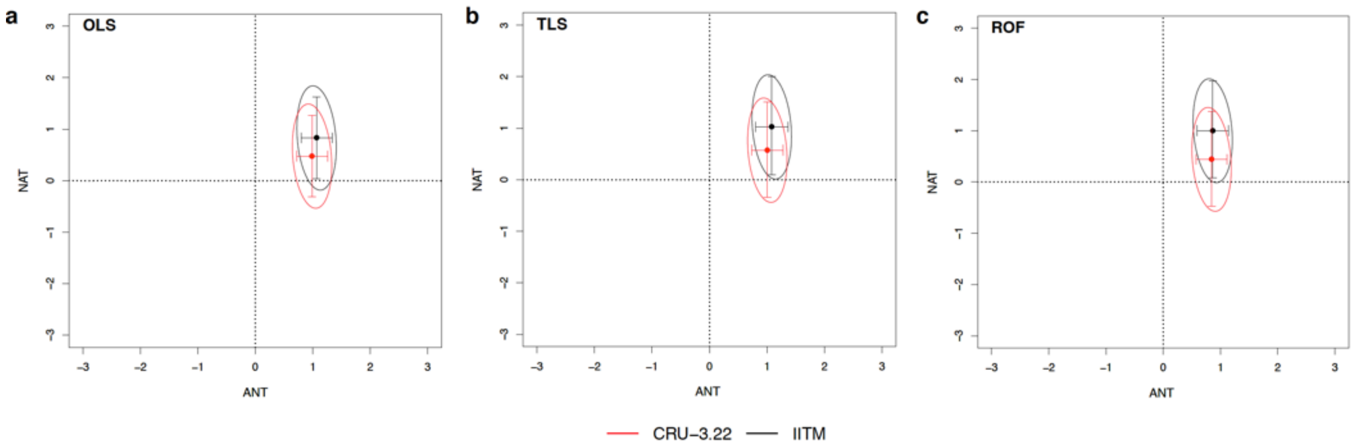

Figure S2. Amplitudes with 5-95% marginal confidence intervals (bars) and 90% confidence regions (ellipses) for ANT vs NAT derived from two-signal analysis using **a** OLS, **b** TLS, and **c** ROF methods using decadal anomalies of annual mean for 1956-2005 period over "All Region". The red and black colours indicate the analysis using CRU 3.22 and IITM datasets respectively.

S3a

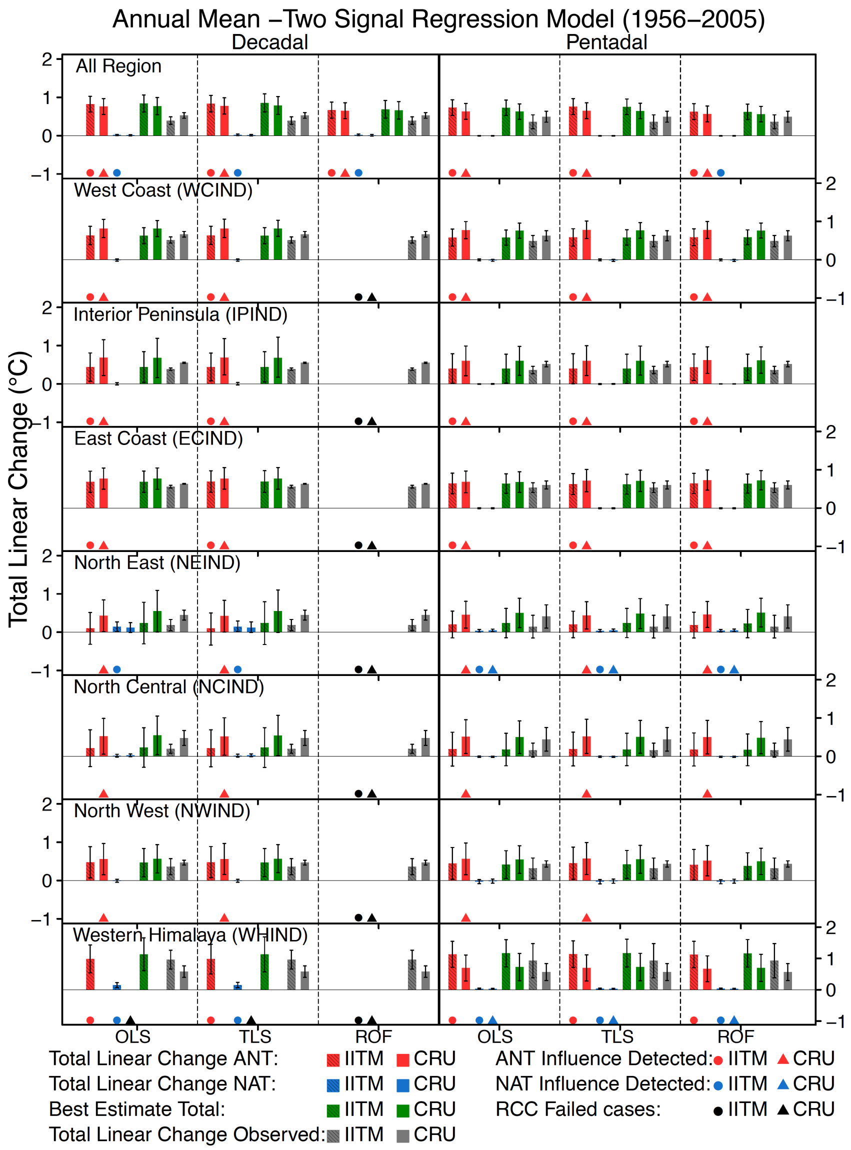

Annual Mean −Two Signal Regression Model (1956−2005)
Decadal
Pentadal
Total Linear Change (°C)
All Region
West Coast (WCIND)
Interior Peninsula (IPIND)
East Coast (ECIND)
North East (NEIND)
North Central (NCIND)
North West (NWIND)
Western Himalaya (WHIND)
OLS
TLS
ROF
OLS
TLS
ROF
Total Linear Change ANT: IITM CRU
Total Linear Change NAT: IITM CRU
Best Estimate Total: IITM CRU
Total Linear Change Observed: IITM CRU
ANT Influence Detected: IITM CRU
NAT Influence Detected: IITM CRU
RCC Failed cases: IITM CRU

S3b

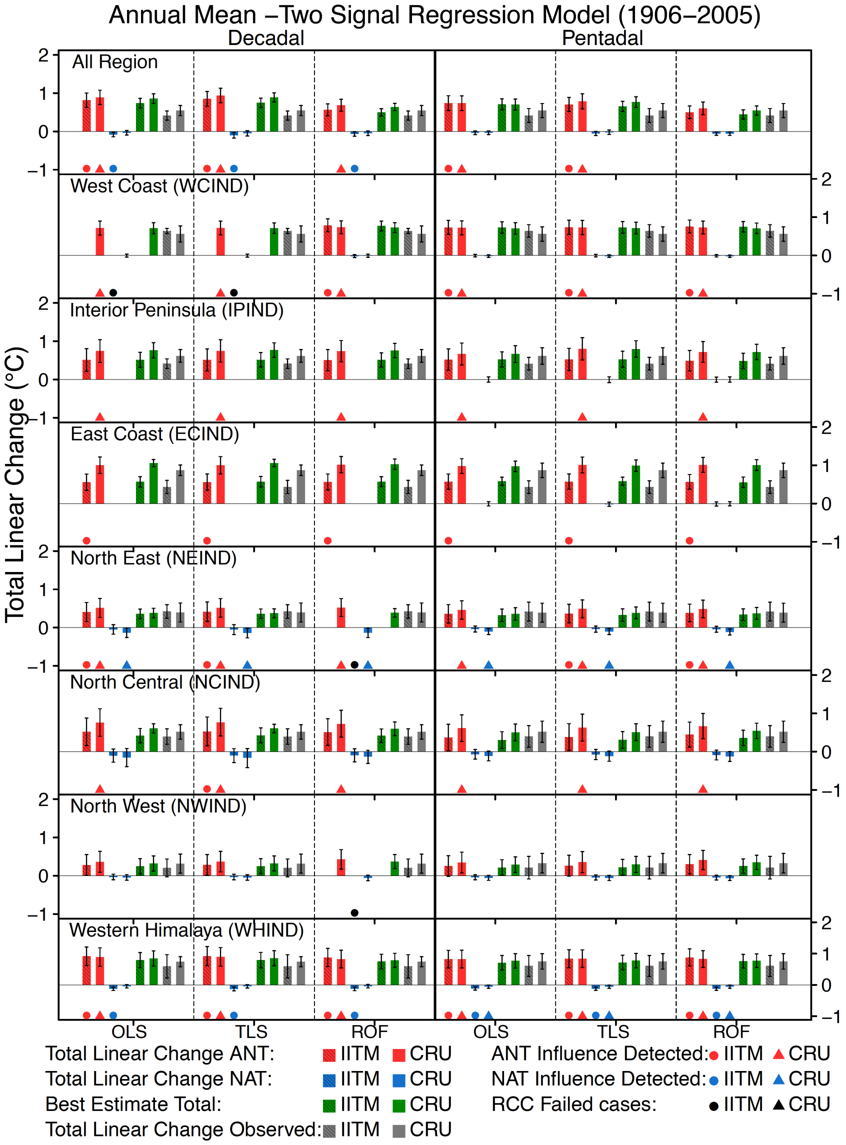

Annual Mean –Two Signal Regression Model (1906–2005)
Decadal
Pentadal
Total Linear Change (°C)
All Region
West Coast (WCIND)
Interior Peninsula (IPIND)
East Coast (ECIND)
North East (NEIND)
North Central (NCIND)
North West (NWIND)
Western Himalaya (WHIND)
OLS
TLS
ROF
Total Linear Change ANT: IITM CRU
Total Linear Change NAT: IITM CRU
Best Estimate Total: IITM CRU
Total Linear Change Observed: IITM CRU
ANT Influence Detected: IITM CRU
NAT Influence Detected: IITM CRU
RCC Failed cases: IITM CRU

S3c

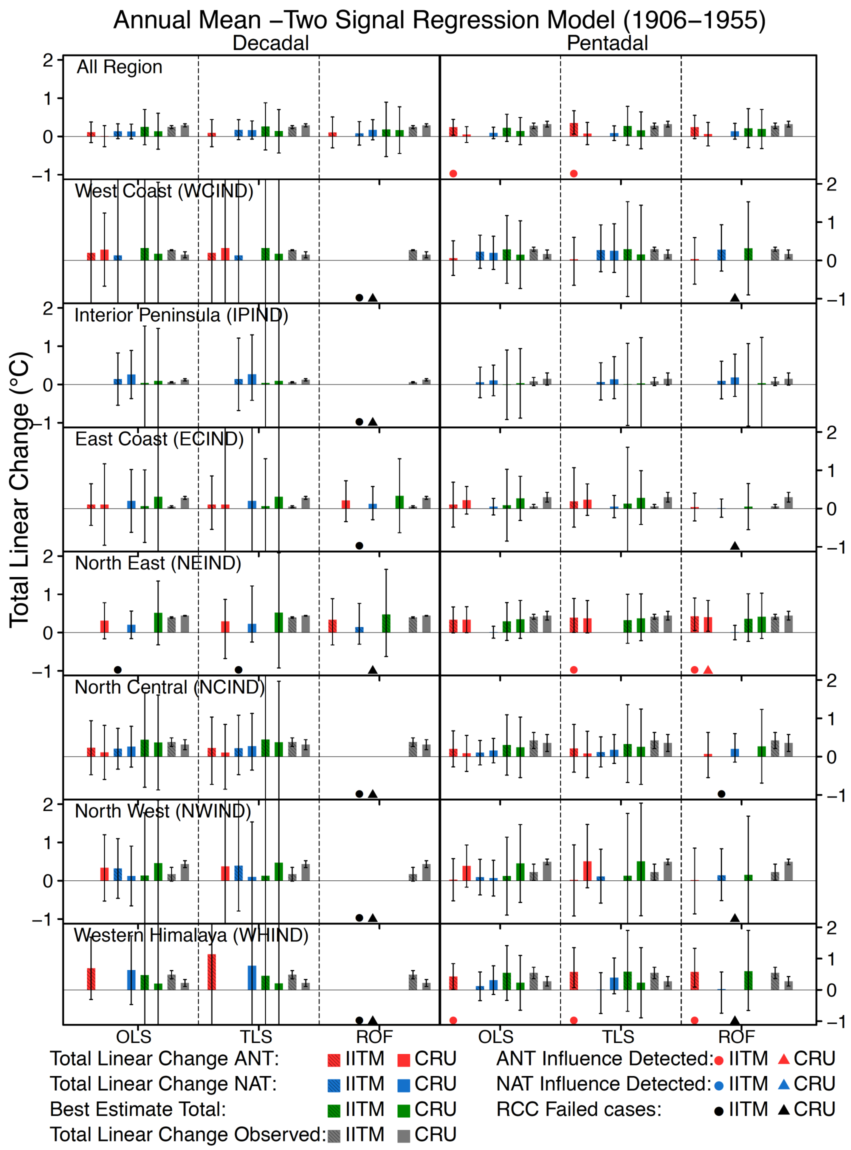

Figure S3. The total linear change of observed and scaled reconstructed simulated forcings of TAS using signal amplitude (β) from OLS, TLS and ROF methods for Decadal and Pentadal analysis of Annual Mean TAS for "All Region" and the seven homogeneous regions for **a** 1956-2005, **b** 1906-2005, and **c** 1906-1955. The hatched bars and non-hatched bars represent the analysis using IITM and CRU as observed dataset respectively. Red(ANT), blue(NAT), green(Best Estimate), and gray(Observed) bars represent the total linear change of simulated response and observations. The cases where effects of ANT (NAT) forcings were detected are marked using red (blue) symbols with circles representing IITM dataset and triangles representing CRU. The black symbols represent cases where the residual consistency check (RCC) failed. Where bars are missing, either the RCC fails for the corresponding signal or there was no detection ($\beta < 0$). Observed trend uncertainties (5-95%) were calculated as in supplementary ref. 5.

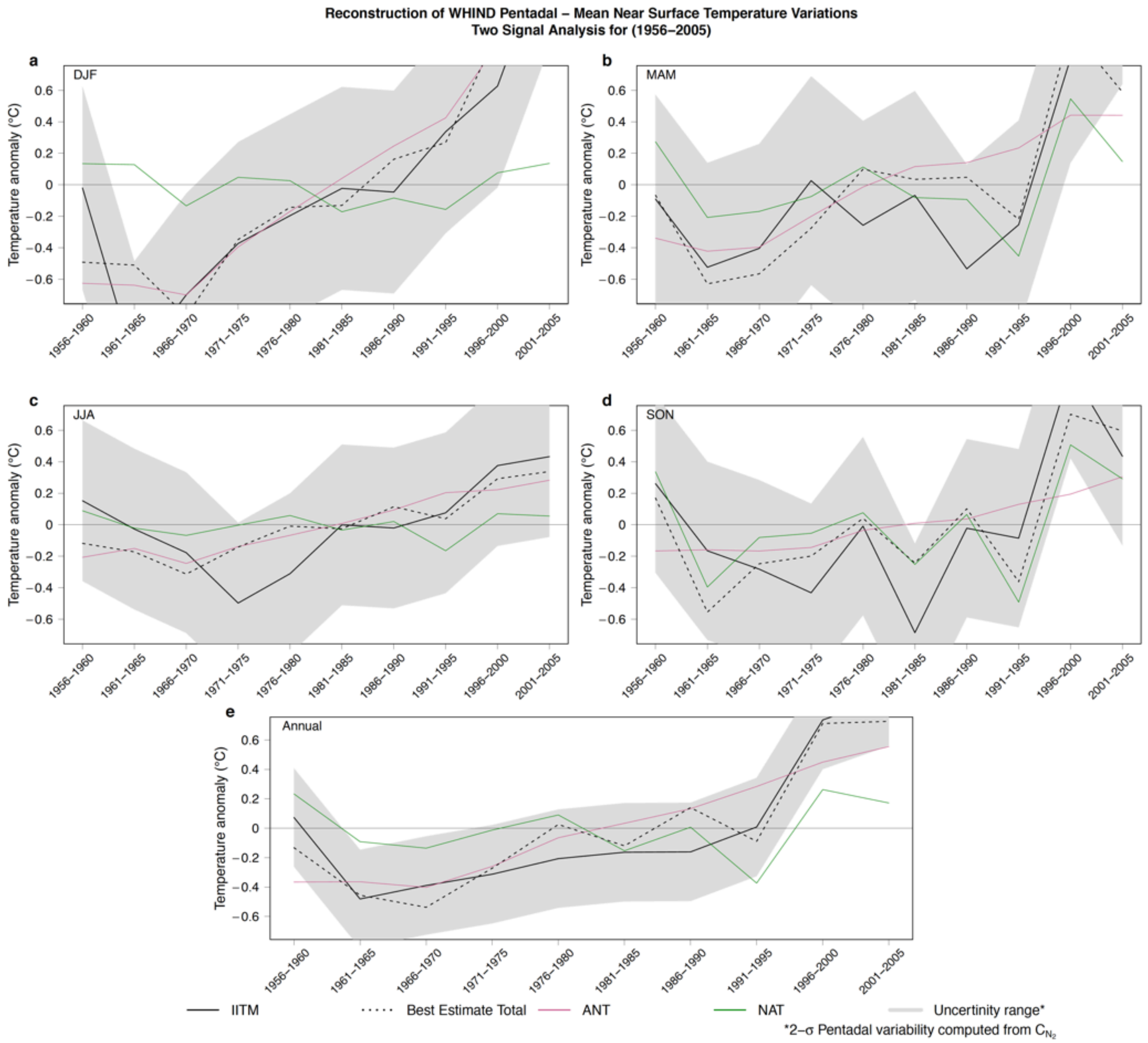

Figure S4. The OLS best estimate reconstruction of pentadal temperature variations during 1956-2005 for **a** DJF, **b** MAM, **c** JJA, **d** SON, and **e** Annual seasons in the WHIND region (black dashed line) shown along with IITM observed dataset (solid black line), and best-estimate contributions from ANT (magenta) and NAT (green). Shaded region centered on the observations shows the uncertainty range due to internal variability (two-sigma pentadal variability computed from C_{N2}).

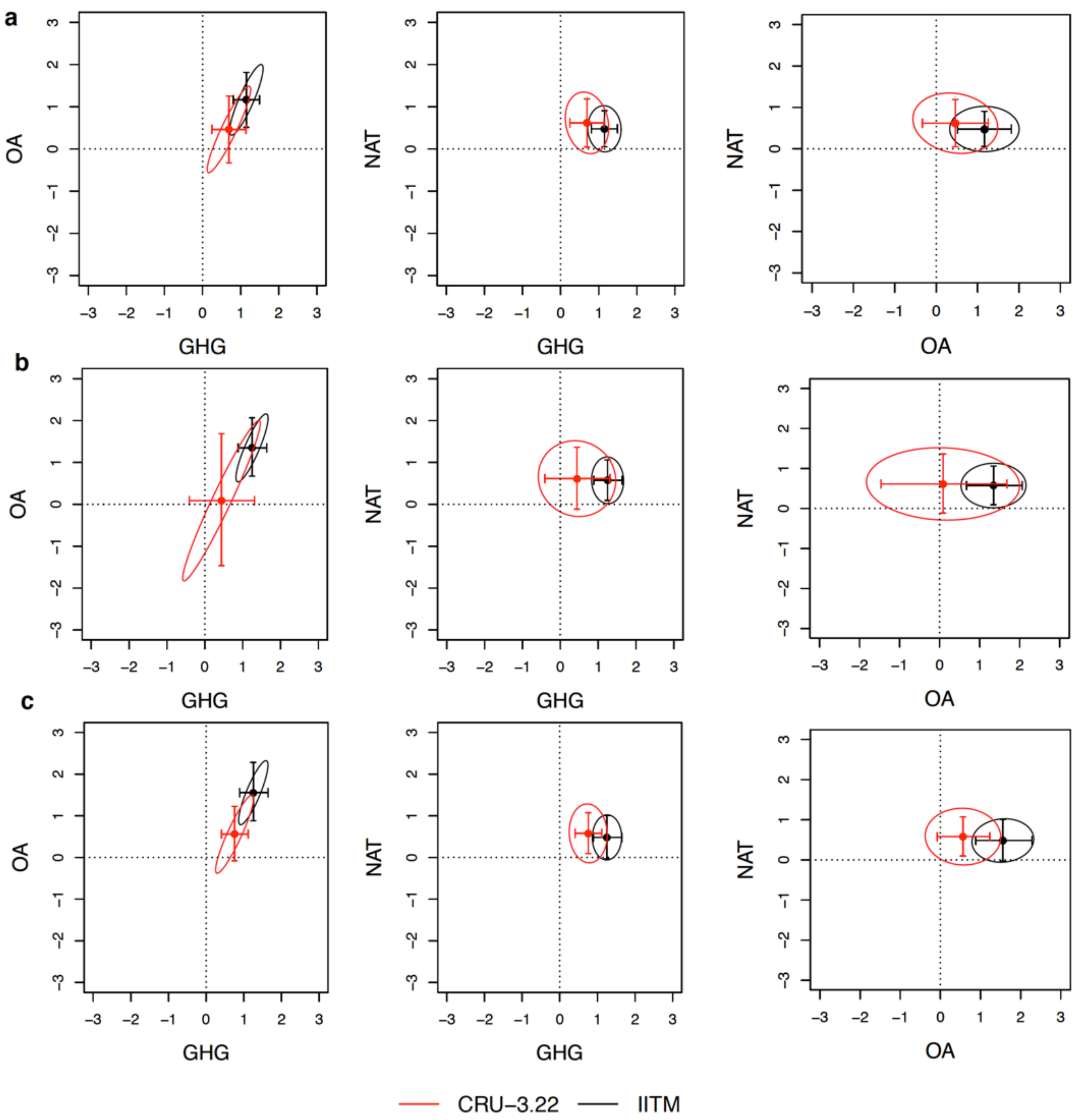

Figure S5. Amplitudes with 5-95% marginal confidence intervals (bars) and 90% confidence regions (ellipses) derived from three-signal **a** OLS, **b** TLS, and **c** ROF analysis GHG vs OA, GHG vs NAT and OA vs NAT using pentadal anomaly of annual mean for 1956-2005 period for “All Region”. The red and black colours indicate the analysis using CRU and IITM datasets respectively.

S6a

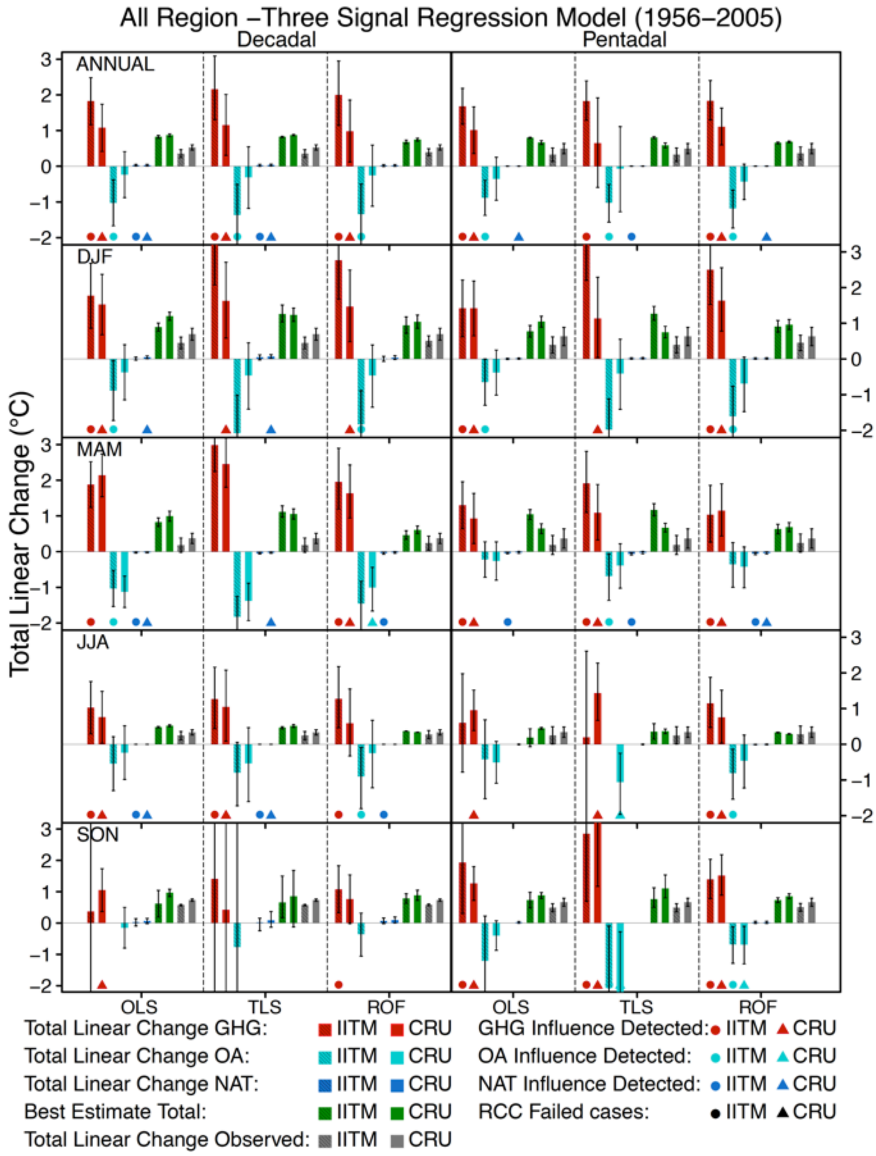
All Region –Three Signal Regression Model (1956–2005)
Decadal
Pentadal
ANNUAL
DJF
MAM
JJA
SON
Total Linear Change (°C)
OLS
TLS
ROF
OLS
TLS
ROF
Total Linear Change GHG: IITM CRU
Total Linear Change OA: IITM CRU
Total Linear Change NAT: IITM CRU
Best Estimate Total: IITM CRU
Total Linear Change Observed: IITM CRU
GHG Influence Detected: IITM CRU
OA Influence Detected: IITM CRU
NAT Influence Detected: IITM CRU
RCC Failed cases: IITM CRU

S6b

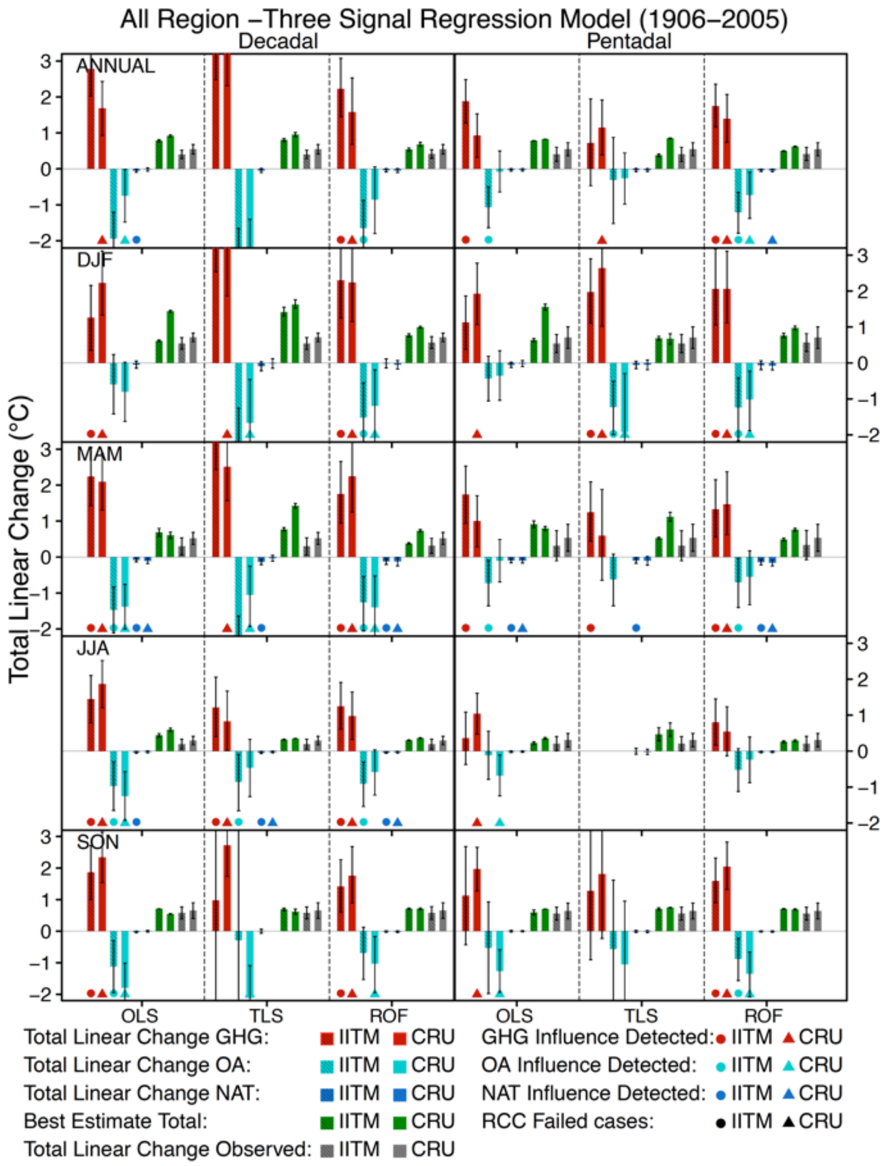
All Region –Three Signal Regression Model (1906–2005)
Decadal
Pentadal
ANNUAL
DJF
MAM
JJA
SON
Total Linear Change (°C)
OLS
TLS
ROF
Total Linear Change GHG: IITM CRU
Total Linear Change OA: IITM CRU
Total Linear Change NAT: IITM CRU
Best Estimate Total: IITM CRU
Total Linear Change Observed: IITM CRU
GHG Influence Detected: IITM CRU
OA Influence Detected: IITM CRU
NAT Influence Detected: IITM CRU
RCC Failed cases: IITM CRU

S6c

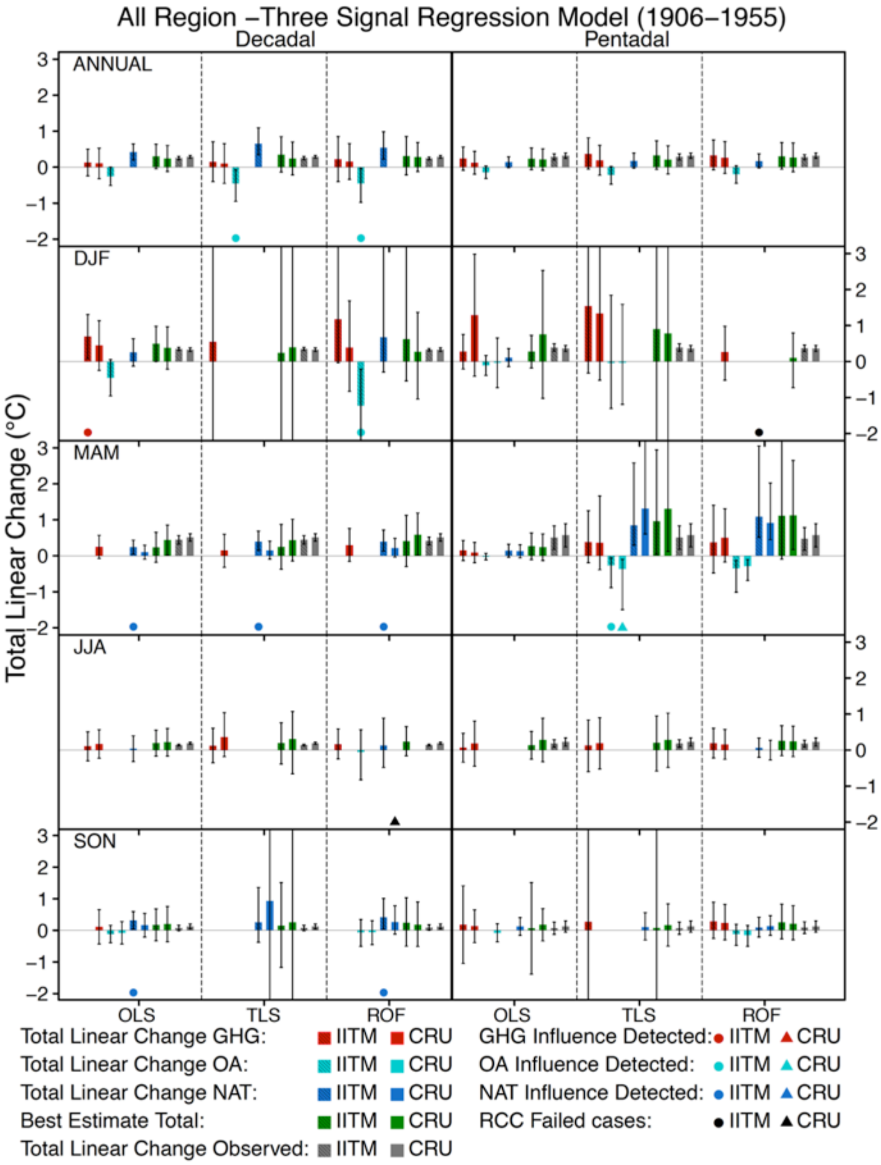

Figure S6. The three-signal total linear change of observed and scaled reconstructed simulated forcings of TAS for "All Region" using best signal amplitude (β) from OLS, TLS and ROF methods for Pentadal and Decadal analysis of annual and seasonal means over **a** 1956-2005, **b** 1906-2005, and **c** 1906-1955. The hatched bars and non-hatched bars represent the analysis using IITM and CRU as observed dataset respectively. Red(GHG), cyan (OA), blue(NAT), green(Best Estimate), and gray(Observed) bars represent the total linear change of simulated response and observations. The cases where effects of individual forcings were detected are marked using colored symbols with circles representing IITM dataset and triangles representing CRU. The black symbols represent cases where the residual consistency check (RCC) failed. Where bars are missing, either the RCC fails for the corresponding signal or there was no detection ($\beta < 0$). Observed trend uncertainties (5-95%) were calculated as in supplementary ref. 5.

S7a

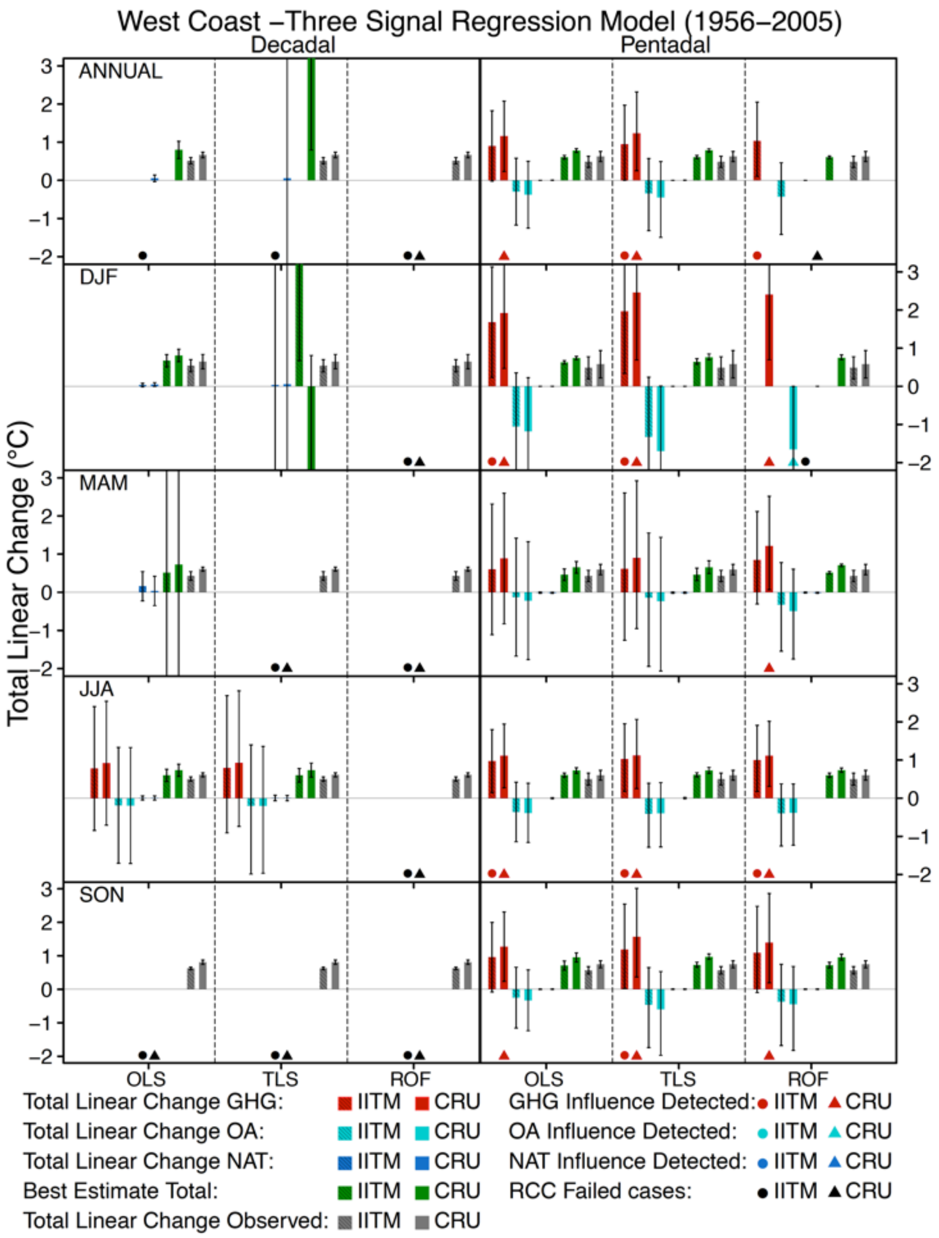

West Coast –Three Signal Regression Model (1956–2005)
Decadal
Pentadal
ANNUAL
DJF
MAM
JJA
SON
Total Linear Change (°C)
OLS
TLS
ROF
OLS
TLS
ROF
Total Linear Change GHG: IITM CRU
Total Linear Change OA: IITM CRU
Total Linear Change NAT: IITM CRU
Best Estimate Total: IITM CRU
Total Linear Change Observed: IITM CRU
GHG Influence Detected: IITM CRU
OA Influence Detected: IITM CRU
NAT Influence Detected: IITM CRU
RCC Failed cases: IITM CRU

S7b

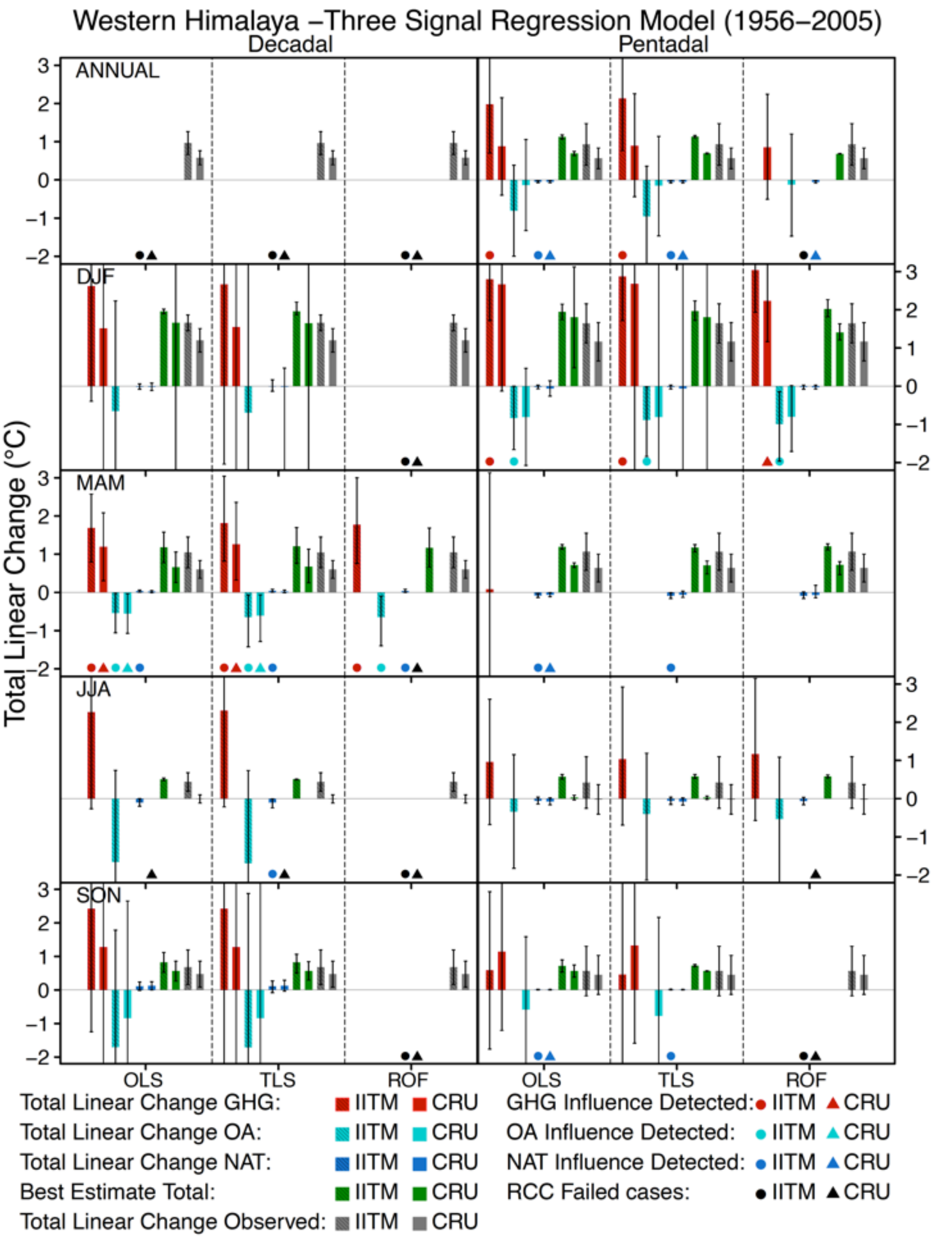

Figure S7. The three-signal total linear change of observed and scaled reconstructed simulated forcings of TAS using best signal amplitude (β) from OLS, TLS and ROF methods for Pentadal and Decadal analysis of annual and seasonal means over 1956 - 2005 period for **a** West Coast (WCIND) and **b** Western Himalayan (WHIND) regions. The hatched bars and non-hatched bars represent the analysis using IITM and CRU as observed dataset respectively. Red(GHG), cyan (OA), blue(NAT), green(Best Estimate), and gray(Observed) bars represent the total linear change of simulated response and observations. The cases where effects of individual forcings were detected are marked using colored symbols with circles representing IITM dataset and triangles representing CRU. The black symbols represent cases where the residual consistency check (RCC) failed. Where bars are missing, either the RCC fails for the corresponding signal or there was no detection ($\beta < 0$). Observed trend uncertainties (5-95%) were calculated as in supplementary ref. 5.

S8-I

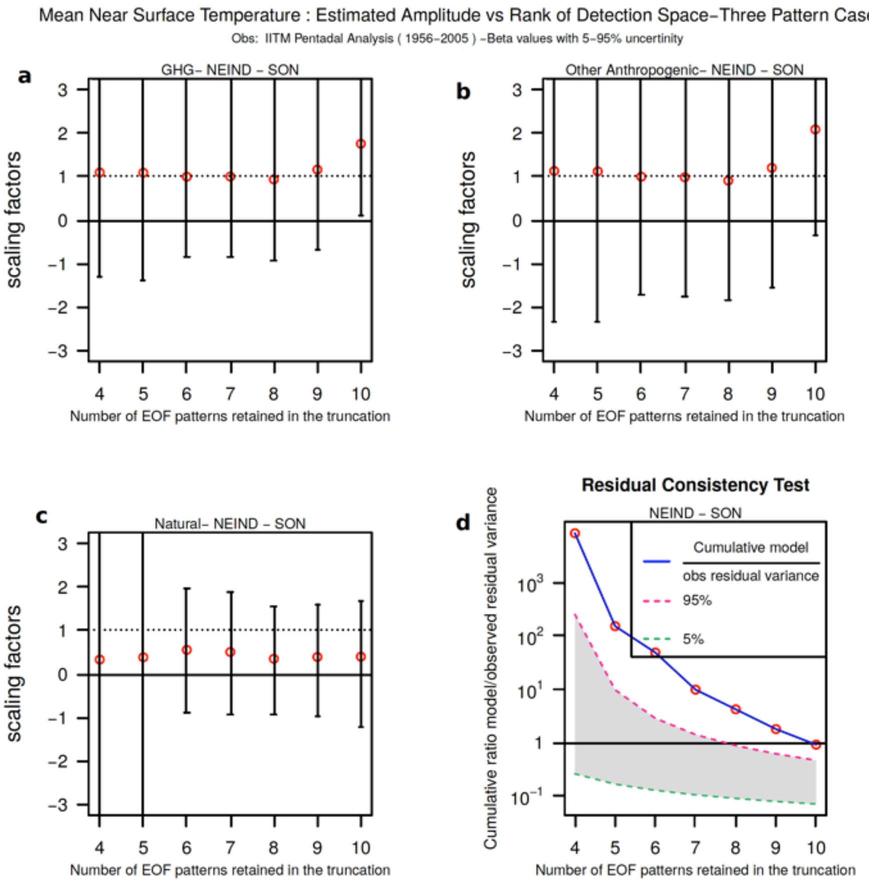

Mean Near Surface Temperature : Estimated Amplitude vs Rank of Detection Space−Three Pattern Case
Obs: IITM Pentadal Analysis (1956−2005) −Beta values with 5−95% uncertinity
a
GHG− NEIND − SON
scaling factors
Number of EOF patterns retained in the truncation
b
Other Anthropogenic− NEIND − SON
scaling factors
Number of EOF patterns retained in the truncation
c
Natural− NEIND − SON
scaling factors
Number of EOF patterns retained in the truncation
d
Residual Consistency Test
NEIND − SON
Cumulative model
obs residual variance
95%
5%
Cumulative ratio model/observed residual variance
Number of EOF patterns retained in the truncation

S8-II

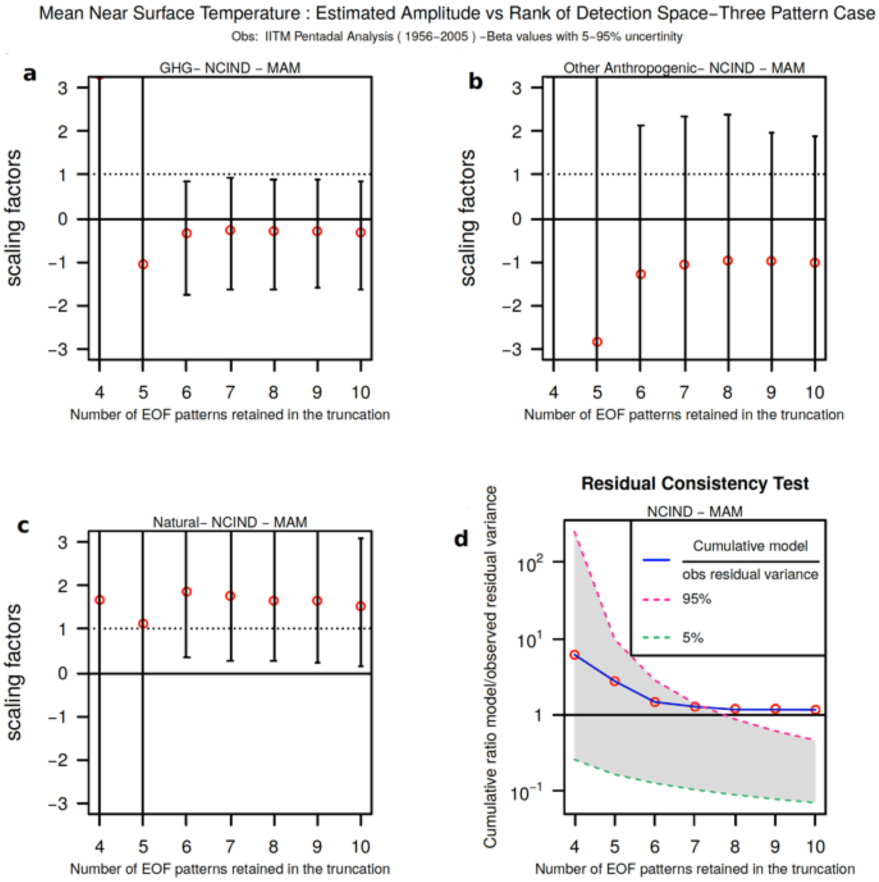

Figure S8. The Residual Consistency Check (RCC) for **I** pentadal SON season analysis in the NEIND region and **II** pentadal MAM season analysis in the NCIND region over 1956-2005 period and using three signal TLS method. The red circles and whiskers in sub-figures **a**, **b**, and **c** of both I and II represent the “best estimate” and the 5 - 95% range for the estimated amplitude of GHG, OA, and NAT forcings respectively for each number of EOFs retained in the truncation. The red and green dotted lines in panel **d** of I and II represent the 5 -95 % confidence level. The RCC fails in I, and passes in II with maximum EOF number as 7. For truncations ≤ 7, the Cumulative ratio model/Observed variance (solid blue line) ranges around unity shows that the model variability is consistent with observed, but for greater truncation levels it falls outside the 5-95 % confidence band. The scaling factor corresponding to EOF 7 was selected as the signal amplitude.

S. No.	Model	Horizontal resolution (lat x lon)	Number of Realizations				Length of longest piControl Run (Years)	Forcing Factors Included in Historical Experiments		
			historical	historicalGHG	historicalNat	historicalAnt		Sulfate Indirect	Carbonaceous Aerosol	Land Use Change
1	IPSL-CM5A-LR	1.89 x 3.75	6	6	3	3	1000	Y	Y	Y
2	GFDL-CM3	2 x 2.5	5	3	3	3	500	Y	Y	Y
3	GFDL-ESM2M	1.51 x 2.5	1	1	1	1	500	**N**	Y	Y
4	GISS-E2-H	2 x 2.5	18	5	10	10	780	Y	Y	Y
5	GISS-E2-R	2 x 2.5	25	5	10	10	1163	Y	Y	Y
6	CCSM4	0.94 x 1.25	8	2	1	4	501	**N**	Y	Y
7	CSIRO-Mk3-6-0	1.84 x 1.87	10	5	5	5	500	Y	Y	**N**
**Total**			**73**	**27**	**33**	**36**				

Table S1. CMIP5 models[4] used in Optimal Fingerprint analysis. Model names, data resolution, number of initial condition ensemble members for each experiment and number of years available for each model's pre-industrial control run also listed. The last column shows the forcing factor accounted for in addition the model runs. Y indicates factor included and N indicates factor not included.

**Region**	WCIND	ECIND	IPIND	NCIND	NWIND	NEIND	WHIND
**Area Weights**	0.08	0.11	0.21	0.18	0.17	0.16	0.09

Table S2. Area weights for the different homogeneous zones used to compute signal amplitude of "All Regions".

Period	Ref. Data	Decadal					Pentadal				
		Observed		OLS	TLS	ROF	Observed		OLS	TLS	ROF
1956-2005	IITM	(0.30 , 0.49)	B. Est.	(0.79, 0.87)	(0.8, 0.84)	(0.64, 0.74)	(0.18 , 0.54)	B. Est.	(0.79, 0.81)	(0.78, 0.84)	(0.63, 0.68)
			OA	(-1.67, -0.38)	(-2.3, -0.51)	(-2.26, -0.5)		OA	(-1.38, -0.39)	(-1.56, -0.51)	(-1.73, -0.67)
			NAT	(0.00, 0.05)	(0.00, 0.06)	(-0.01, 0.05)		NAT	(0.00, 0.01)	(0.00, 0.01)	(0.00, 0.01)
			GHG	(1.16, 2.48)	(1.31, 3.09)	(1.15, 2.95)		GHG	(1.18, 2.18)	(1.29, 2.39)	(1.30, 2.40)
	CRU	(0.45 , 0.60)	B. Est.	(0.83, 0.91)	(0.86, 0.90)	(0.71, 0.79)	(0.35 , 0.64)	B. Est.	(0.61, 0.72)	(0.52, 0.65)	(0.66, 0.71)
			OA	(-0.88, 0.41)	(-1.18, 0.54)	(-1.12, 0.59)		OA	(-0.95, 0.25)	(-1.28, 1.11)	(-0.93, 0.06)
			NAT	(0.00, 0.05)	(0.01, 0.06)	(0.00, 0.05)		NAT	(0.00, 0.01)	(0.00, 0.01)	(0.00, 0.01)
			GHG	(0.42, 1.74)	(0.31, 2.01)	(0.12, 1.86)		GHG	(0.34, 1.67)	(-0.59, 1.92)	(0.60, 1.63)
1906-2005	IITM	(0.29 , 0.53)	B. Est.	(0.75, 0.81)	(0.77, 0.84)	(0.50, 0.58)	(0.23 , 0.60)	B. Est.	(0.78, 0.78)	(0.35, 0.42)	(0.49, 0.51)
			OA	(-2.67, -1.21)	(-3.65, -1.66)	(-2.49, -0.87)		OA	(-1.64, -0.50)	(-1.52, 0.88)	(-1.79, -0.65)
			NAT	(-0.10, -0.01)	(-0.10, 0.01)	(-0.09, 0.00)		NAT	(-0.06, 0.01)	(-0.08, 0.01)	(-0.07, 0.00)
			GHG	(2.03, 3.53)	(2.49, 4.52)	(1.45, 3.07)		GHG	(1.28, 2.48)	(-0.47, 1.95)	(1.16, 2.35)
	CRU	(0.41 , 0.68)	B. Est.	(0.89, 0.95)	(0.90, 1.01)	(0.63, 0.74)	(0.36 , 0.73)	B. Est.	(0.83, 0.83)	(0.84, 0.86)	(0.61, 0.64)
			OA	(-1.48, -0.01)	(-3.71, -1.41)	(-1.8, 0.05)		OA	(-0.64, 0.5)	(-0.98, 0.44)	(-1.38, -0.09)
			NAT	(-0.06, 0.03)	(NA, NA)	(-0.10, 0.01)		NAT	(-0.06, 0.01)	(-0.07, 0.01)	(-0.08, -0.01)
			GHG	(0.93, 2.43)	(2.32, 4.63)	(0.68, 2.53)		GHG	(0.32, 1.53)	(0.39, 1.92)	(0.74, 2.07)
1906-1955	IITM	(0.21 , 0.28)	B. Est.	(-0.04, 0.64)	(-0.14, 0.85)	(-0.21, 0.85)	(0.20 , 0.35)	B. Est.	(-0.07, 0.53)	(-0.06, 0.73)	(-0.06, 0.68)
			OA	(-0.51, 0.00)	(-0.95, -0.08)	(-0.98, -0.04)		OA	(-0.32, 0.04)	(-0.47, 0.02)	(-0.45, 0.04)
			NAT	(0.20, 0.65)	(0.34, 1.09)	(0.22, 0.98)		NAT	(-0.01, 0.29)	(-0.03, 0.39)	(-0.02, 0.37)
			GHG	(-0.24, 0.50)	(-0.40, 0.70)	(-0.40, 0.85)		GHG	(-0.09, 0.56)	(-0.05, 0.81)	(-0.08, 0.76)
	CRU	(0.25 , 0.33)	B. Est.	(-0.12, 0.60)	(-0.22, 0.70)	(-0.12, 0.69)	(0.25 , 0.40)	B. Est.	(-0.09, 0.51)	(-0.19, 0.60)	(-0.13, 0.67)
			OA	(NA, NA)	(NA, NA)	(NA, NA)		OA	(NA, NA)	(NA, NA)	(NA, NA)
			NAT	(NA, NA)	(NA, NA)	(NA, NA)		NAT	(NA, NA)	(NA, NA)	(NA, NA)
			GHG	(-0.32, 0.53)	(-0.45, 0.65)	(-0.34, 0.66)		GHG	(-0.19, 0.44)	(-0.22, 0.61)	(-0.17, 0.71)

Table S3. Observed and attributable trends (°C per period length given as 5–95% ranges) over "All Region" from the three-signal analysis of TAS change. Results are shown for the three periods analysed 1956-2005, 1906-2005, and 1906-1955. The GHG, OA, and NAT attributable trends are shown along with the Best Estimate from the three forcings. Observed trends and 5-95% confidence interval are calculated as in Santer et al 2000 (ref. 5). The cells marked (NA, NA) represent cases where the residual consistency check (RCC) fails or there was no detection ($\beta < 0$).

Supplementary Text

1. Band-pass filter details:

We applied a band-pass filter used in Santer et al., 2011 (ref. 6) to observed and modelled detrended TAS monthly anomalies in order to compare the observed and modelled variabilities in the frequencies of interest. The filtering allows us to focus on variability on 10-year timescales, with half power points at 5 and 20 years.

2. Processing of data:

The near surface temperature (TAS) from observations and CMIP5 simulations are processed as described below.

2.1 The observation vector Y is processed as follows: The CRU observed dataset regridded to a 1x1 grid and masked over each homogeneous temperature zone and spatially averaged to produce a monthly time series for each homogeneous region. This step is not required for the IITM data since it already comes as a time series for the region. We then compute the seasonal average(annual, DJF, MAM, JJA & SON) of above datasets. Then we compute decadal and pentadal averages from each seasonal mean time series. The decadal and pentadal anomalies are centered by removing the mean for the period under consideration (1906-1955, 1906-2005, 1956-2005)

2.2 The simulation vector X is produced from the individual forcings runs as follows: First compute for each ensemble member of each model experiment the monthly spatial average over each homogeneous region after regridding and masking as with the CRU data. Then calculate the multi-model ensemble (MME) average time series by first taking the ensemble average for each model then calculating the mean across models. Compute the seasonal mean (Annual, DJF, MAM, JJA, and SON) of MME average time series. Compute the decadal (pentadal) average of each seasonal mean of MME average. The decadal and pentadal averaged data were centered temporally by removing the mean over full period (the full length of simulation for piControl; 1906-2005 for all other experiments). We conducted the optimal fingerprint analysis over all regions by concatenating the area weighted (Table S2) data of all seven homogeneous regions (denoted "All Regions"). For the "All Region" case the temporal centering carried out after the concatenation of area weighted datasets . We denote the resulting vector as X_{ANT} (for MME of the historicalAnthro simulation), X_{NAT} (for historicalNat simulation), X_{GHG} (for historicalGHG) etc. The columns of X were given by X_{ANT} & X_{NAT} for two signal case. For the three signal case we used a linear transformation A for

deriving the X' (a linear combination of GHG, OA, and NAT) from X (a linear combination of historicalGHG, historical, and historicalNat).

$$X' = X * A$$
$$(GHG, OA, NAT) = (historicalGHG, historical, historicalNat) * A$$
$$= (historicalGHG, historical, historicalNat)\begin{bmatrix}1 & -1 & 0\\ 0 & 1 & 0\\ 0 & -1 & 1\end{bmatrix}$$

Then the corresponding scaling factors for (GHG, OA, NAT) and $(historicalGHG, historical, historicalNat)$ are β' and β respectively.

$$X'\ \beta' = X\beta$$
$$(XA)\beta' = X\beta$$

Then

$$\beta' = A^{-1}\beta$$

Therefore

$$\begin{pmatrix}\beta_{GHG}\\ \beta_{OA}\\ \beta_{NAT}\end{pmatrix} = \begin{pmatrix}1 & 1 & 0\\ 0 & 1 & 0\\ 0 & 1 & 1\end{pmatrix}\begin{pmatrix}\beta_{\text{historicalGHG}}\\ \beta_{\text{historical}}\\ \beta_{\text{historicalNat}}\end{pmatrix}$$

2.3 The two noise covariance matrices C_{N1} & C_{N2} were estimated from piControl dataset as follows:

Given that the different models have piControl runs of varying lengths, we extracted 500 years (length of the shortest piControl run available) from the end of the piControl run available from each model. We then calculated the spatial average over each homogeneous region as with the individual forcing runs. Then we compute the seasonal averages (Annual, DJF, MAM, JJA, and SON) followed by decadal and pentadal means. We then extract all 10-decade long segments that overlap by all but 1 decade. We get 40 segments from each model's piControl time series of which, we choose the first 20 segments for constructing C_{N1} and the latter 20 segments for C_{N2}. We repeat the above procedure for each model's piControl adding rows to C_{N1} & C_{N2} resulting in 140 rows in each of the noise matrices. This procedure utilizes piControl runs from all the models yielding a common EOF basis as in Jones et al., 2013 (ref. 7).

3. Truncation level and Residual Consistency Check (RCC):

Increasing the truncation level (EOF number) will introduce unrealistically low variance. Similarly if choosing small truncation level will give uncertainty estimate are unreliable. Before drawing any further conclusions, therefore, we need to establish the maximum truncation at which the model is reliable. The residual consistency check tests the hypothesis that model simulated internal variability

is equal to observed.

Residual consistency check for OLS & TLS method:

The F- test based on ratio of model internal variance to observed residual variance is used. The red dots in Fig. S8 I & II (**a**, **b**, **c**) show the evolution of $(\kappa - m)/r^2$, where κ, is the number of EOFs and m is the pattern number, and r^2 is the residual (r^2 follows $(\kappa - m)F_{(\kappa-m,\nu)}$ distribution, where ν denotes the degrees of freedom). Therefore $(\kappa - m)/r^2$ follows the $\frac{1}{F_{(\kappa-m,\nu)}}$distribution. This quantity is interpreted physically as 'cumulative ratio of model/observed residual variance'. The green and red dashed lines represent the 5 -95 % confidence level respectively. Then its corresponding scaling factors were selected as the signal amplitude. The amplitude factors depends on the truncation number (number of EOF used). When less number of EOF used then it fails to capture the overall variability of observed and simulated pattern. But if more number of EOFs are used, then it may add more noise to the signal. The RCC can give suitable truncation values. In Fig. S8, RCC fails in I and passes in II for maximum number of EOFs equal to 7. Refer Allen and Tett 1999(ref 8) and Allen and Stott 2003 (ref 9)

Residual consistency check for ROF method [10]:

Here we employed the RCC used in TLS method. TLS regression model is based on the SVD of the concatenated matrix $M = [Y, X]$, which is an $n \times (l + 1)$ matrix. Let λ_i's are the eigenvalues of M in decreasing order and v_i's are its corresponding eigenvectors (where i varies from 1 to (l+1)). The RCC check is based on$\lambda_{min}^2 = \lambda_{l+1}^2$(Equation 35 in ref. 9). The ROF method uses Monte Carlo simulations (N=10000) for estimating the null distributions of the RCC. We assume that the scaling factors failed the RCC, if its p-value exceed 10% significance level.

Supplementary References

1. Taylor, K. E. Summarizing multiple aspects of model performance in a single diagram. *J. Geophys. Res.* **106**, 7183-7192 (2001).
2. Kothawale, D. R. & Rupa Kumar, K. On the recent changes in surface temperature trends over india. *Geophys. Res. Lett.* **32**, L18714+ (2005).
3. Harris, I., Jones, P. D., Osborn, T. J. & Lister, D. H. Updated high-resolution grids of monthly climatic observations – the CRU TS3.10 dataset. *Int. J. Climatol.* **34**, 623-642 (2014).
4. Taylor, K. E., Stouffer, R. J. & Meehl, G. A. An overview of CMIP5 and the experiment design.

Bull. Amer. Meteor. Soc. **93**, 485-498 (2011).

5. Santer, B. D. *et al.* Statistical significance of trends and trend differences in layer-average atmospheric temperature time series. *J. Geophys. Res.* **105**, 7337-7356 (2000).
6. Santer, B. D. et al. Separating signal and noise in atmospheric temperature changes: The importance of timescale. *J. Geophys. Res. D*: Atmos. **116**, (2011).
7. Jones, G. S., Stott, P. A. & Christidis, N. Attribution of observed historical near-surface temperature variations to anthropogenic and natural causes using CMIP5 simulations. *J. Geophys. Res. D*: Atmos. **118**, 4001–4024 (2013).
8. Allen, M. R. & Tett, S. F. B. Checking for model consistency in optimal fingerprinting. *Clim. Dyn.* **15,** 419–434 (1999).
9. Allen, M. R. & Stott, P. A. Estimating signal amplitudes in optimal fingerprinting, part I: theory. *Clim. Dyn.* **21,** 477–491 (2003).
10. Ribes, A., Planton, S. & Terray, L. Application of regularised optimal fingerprinting to attribution. Part I: method, properties and idealised analysis. *Clim. Dyn*. **41**, 2817–2836 (2013).
